# Photothermal Temperature-Modulated Cancer Metastasis Harnessed Using Proteinase-Triggered Assembly of Near-Infrared II Photoacoustic/Photothermal Nanotheranostics

**DOI:** 10.1021/acsami.4c07173

**Published:** 2024-07-24

**Authors:** Yao-Chen Chuang, Yu Hsia, Chia-Hui Chu, Sivasubramanian Maharajan, Fang-Chi Hsu, Hsin-Lun Lee, Jeng Fong Chiou, Hui-Ju Ch’ang, Lun-De Liao, Leu-Wei Lo

**Affiliations:** † Institute of Biomedical Engineering and Nanomedicine, National Health Research Institutes, Zhunan, Miaoli 35053, Taiwan; ‡ Department of Radiation Oncology, Taipei Medical University Hospital, Taipei 110301, Taiwan; § Institute of Biotechnology, National Tsing Hua University, Hsinchu 30013, Taiwan; ∥ The Ph.D. Program for Translational Medicine, College of Medical Science and Technology, Taipei Medical University and Academia Sinica, Taipei 110301, Taiwan; ⊥ Department of Radiology, School of Medicine, College of Medicine, Taipei Medical University, Taipei 110301, Taiwan; # National Institute of Cancer Research, National Health Research Institutes, Zhunan, Miaoli 35053, Taiwan

**Keywords:** plasmonic photothermal therapy, gold nanodandelions, photoacoustic imaging, matrix metalloproteinases, near-infrared window I and II, metastasis

## Abstract

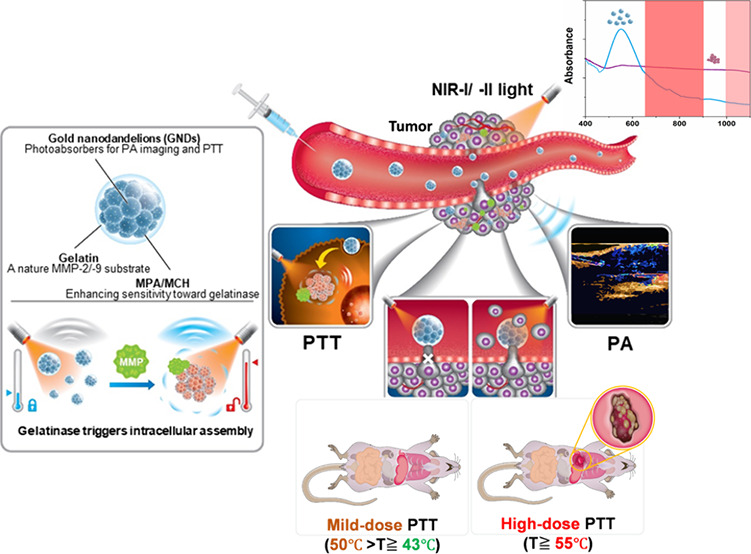

Here we demonstrate that cancer metastasis could be modulated by the judicious tuning of physical parameters such as photothermal temperature in nanoparticle-mediated photothermal therapy (PTT). This is supported by theranostic nanosystem design and characterization, *in vitro* and *in vivo* analyses, and transcriptome-based gene profiling. In this work, the highly efficient near-infrared II (NIR-II) photoacoustic image (PA)-guided PTT are selectively activated using our developed matrix metalloproteinase (MMP)-triggered *in situ* assembly of gold nanodandelions (GNDs@gelatin). Unlike other “always-on” NIR PTT agents lacking specific bioactivation and suffering from the intrinsic nonspecific pseudosignals and treatment-related side effects such as metastasis, our GNDs@gelatin possesses important advantages while deployed in cancer PTT that include the following: (1) The theranostic effects could be “turned on” only after specific MMP-2/-9 activity and with acidity in the tumor microenvironment. (2) The quantitative PA diagnosis allows for precise PTT planning for better cancer treatment. (3) GNDs@gelatin could noninvasively quantify MMP activity and efficiently harness NIR-I (808 nm) and NIR-II (1064 nm) energies for tumor ablation. (4) The multibranched nanostructures reabsorb scattered laser photons, thus enhancing the surface plasmons for the pronounced photothermal conversion of aggregated GNDs@gelatin *in situ*. (5) It is noteworthy that *in situ* tumor eradication at higher PTT temperature (>55 °C) mediated by GNDs@gelatin could induce subsequent metastasis, which could be otherwise abolished at lower PTT temperatures (50 °C > *T* > 43 °C). (6) Furthermore, the gene profiling using transcriptome-based microarray including GO and KEGG analyses revealed that 315 differentially expressed genes were identified in higher PTT temperature treated tumors compared with lower PTT temperature ones. These were enriched into some well-known cancer-related pathways, such as cell migration pathway, signal transductions, cell proliferation, wound healing, PPAR signaling, and metabolic pathways. These observations suggest a new perspective of “moderate-is-better” in nanoparticle-mediated PTT for maximizing its therapeutic/prognosis benefits and translational potential with metastasis inhibition.

## Introduction

Over the past several decades, the concept of theranostics, which integrates diagnosis with therapeutic capability in one platform, has attracted great attention in the field of cancer therapy.^1−3^ With the continued development of nanotechnology, today, various multifunctional nanoparticle-imaging techniques, such as magnetic resonance imaging (MRI), computed tomography (CT), positron emission tomography (PET), single-photon emission computed tomography (SPECT), and optical imaging, have been designed to perform real-time image-guided therapy. Among them, optical imaging stands out as one of the most promising approaches for the precise diagnosis of cancer, especially photoacoustic (PA) imaging.^4^ PA imaging, as a new imaging modality, exceeds the optical diffusion limit because it detects phonons instead of photons after light excitation. Indeed, it allows for deep tissue-imaging and possesses higher spatial resolution when compared with traditional optical imaging techniques.^5^ In addition, the signal of PA imaging is related to photothermal conversion, and the principles of selecting contrast agents for PA imaging are naturally consistent with those for photothermal therapy (PTT), which makes photothermal agents highly promising candidates for PA imaging-guided PTT.^6,7^

As one class of strong photothermal nanomaterials, gold-based nanostructures have been widely explored as PA imaging-guided nanoagents due to their high photothermal conversion efficiency. In the past few decades, by controlling size, morphology, and/or surface coating, the working window of gold nanostructures can be shifted into the near-infrared (NIR) range where the penetration of light in tissue is maximized.^8−11^ Moreover, the biocompatibility of gold nanostructures and the ease of surface modification are also attractive characteristics for applications in targeted delivery. Although a variety of gold nanostructures, such as gold nanorods, nanocages, nanoshells, and nanostars, have been demonstrated for photothermal cancer therapy and PA imaging, challenges remain for practical applications. First, the reliable large-scale synthesis of concentrated and high-quality anisotropic nanostructures and their subsequent purification constitute the two main obstacles for them to advance for clinical applications.^12^ Furthermore, a fraction of NIR-absorbing nanomaterials will remain in the surrounding tissue environment and likely be internalized by healthy cells during both active and passive targeted therapeutic approaches. These nonselective triggering, “always on” NIR-absorbing nanomaterials maintain a high signal-to-noise ratio for healthy and diseased tissues, which results in nonspecific heating and their ablation.^13,14^ To overcome these obstacles, numerous strategies have been developed for the synthesis of activatable gold nanoparticles (AuNPs) for turn-on NIR-absorbing nanomaterials.^15−19^ For example, Kim et al. reported pH-responsive AuNPs that can agglomerate at a tumorous site and elicit a significant absorption wavelength shift into the NIR optical window to spare normal cells and minimize unwanted damage.^20^ Furthermore, other stimuli, including extrinsic (light and salt) and intrinsic (oxidative stress and enzyme) approaches, have been reported to induce aggregation of AuNPs at tumorous sites. Nonetheless, assembly of AuNPs is known to increase their scattering efficiency, which results in a decrease in photothermal conversion efficiency. Therefore, a higher dose of PTT-agent or higher laser power density is necessary to achieve the desired effects.

From the clinical perspective, tumor microenvironment (TME)-responsive PTT-agents may be a promising strategy for the treatment of glioma, which is one of the most aggressive and common intracranial tumors. Accumulated evidence indicates that the amounts of matrix metalloproteinases (MMPs) protein, especially MMP-2 and MMP-9, are significantly increased in patients with malignant glioma, particularly in glioblastoma multiforme.^21,22^ Previously, we have developed a facile, large-scale synthesis of multibranched, flower-like AuNPs, i.e., gold nanodandelions (GNDs), using a novel gelatin-directed method and demonstrated significantly enhanced radiosensitization-induced ROS generation and anticancer drug loading.^23^ In this study, we further the equip GND nanoplatform with the theranostic applicability of PA-guided PTT and demonstrate its *in vivo* modulation of induced cancer metastasis. The significant features are described briefly as follows: (1) Gelatin-embedded GNDs (GNDs@gelatin) were constructed as a TME-responsive PTT/PA theranostic agent. (2) Gelatinase (e.g., MMP-2 and MMP-9) exposure in TME promoted the self-assembly of GNDs@gelatin into microstructures with enhanced PA signal for tumor imaging and localized plasmonic heating for photothermal therapeutics. (3) The design allows tumor ablation using heat as a nonchemical treatment that circumvents glioma heterogeneity limitations and conventional drug resistance mechanisms. (4) The MMP-responsive GNDs@gelatin could form intracellular aggregation and turn on their absorption peak position toward NIR-I and II regions. Therefore, assembly of GNDs@gelatin can absorb NIR-II light and convert the absorbed NIR into heat, which should be sufficient to induce deep and localized hyperthermia and achieve tumor destruction. (5) Multibranched GNDs@gelatin were able to reabsorb the scattered light, enhance their photothermal conversion ability, and make the power density of PTT within safety limits. (6) Although hyperthermia could eradicate most primary tumors, PTT engaging at a higher temperature (*T* > 55 °C) exhibited a propensity to induce metastasis *in vivo*, while in the range of lower temperature (50 °C > *T* > 43 °C), no metastasis was incurred. In aggregate, this work constitutes a new conceptual breakthrough in which temperature-dependent PTT is demonstrated to constitute an important approach to modulate tumor metastasis, as well as for glioma theranostics.

## Materials and Methods

### Chemicals and Materials

Hydrogen tetrachloroaurate (III) trihydrate (HAuCl_4_), trisodium citrate (Na_3_C_6_HO_7_), l-ascorbic acid (C_6_H_8_O_6_), type A gelatin (MW 20–200 kDa), recombinant MMP-2, sodium chloride (NaCl), calcium chloride (CaCl_2_), 6-mercaptohexan-1-ol (MCH), and 3-sulfanylpropanoic acid (MPA) were purchased from Sigma-Aldrich. All chemicals were of analytical grade. Nanopure water was obtained by passing twice-distilled water through a Milli-Q system (18 MU cm; Millipore, Bedford, MA, U.S.A.).

### Dual-Modality US/PA Imaging System

The custom-made dark-field dual-modality ultrasound (US) and photoacoustic (PA) (i.e., US/PA) imaging system contained an 18.5-MHz high-frequency US transducer (L22-14 V, Verasonics, USA) and a customized light delivery system with fiber bundles. The received US/PA signals were associated with a multichannel high-frequency US platform (Vantage 128, Verasonics, USA). The laser excitation should be synchronized with the US information obtained for the PA mode imaging. A customized dark-field illumination system with fiber bundles was used to efficiently deliver the laser energy to the region of interest (ROI) and also form a PA dark-field between the focus point and US transducer for a better signal-to-noise ratio (SNR). The PA imaging resolution was estimated based on the full width at half-maximum (fwhm) of each Gaussian function from the signals and was measured to be 124 ± 31 μm for the developed system. The characterization and detailed specifications of the developed US/PA imaging system were reported in our previous studies.^24,25^ The *in vitro* and *in vivo* PA B-scans were analyzed using a custom-made interface based on MATLAB (R2007a, MathWorks, USA). The maximum permissible exposure was well within the American National Standards Institute (ANSI) (i.e., less than 20 mJ·cm^–2^) during the experiment.

### Synthesis of Gold Seed

Au seed nanoparticles were prepared according to the literature. Briefly, 3 mL of 38.8 mM sodium citrate was added to 50 mL of 1 mM HAuCl_4_ solution, and the mixture was heated by microwave irradiation. After 90 s, the mixture acquired a red-purple color, and then the solution was stored at 4 °C prior to further use. A transmission electron microscopy (TEM) examination showed that the resulting AuNPs were spherical in shape with an average diameter of 20 nm.

### Synthesis of GNDs@gelatin

GNDs@gelatin was obtained through a seed-mediated route. Briefly, 100 mL of gelatin solution (10 mg·mL^–1^) was kept at room temperature under gentle stirring. Then, 7.5 mL of citrate-capped gold seeds and 200 μL of 250 mM HAuCl_4_ were added, and this mixture was aged for 15 min. The growth of GNDs@gelatin occurred by adding 2.5 mL of 10 mM ascorbic acid aqueous solution, and stirring was immediately stopped. At the end of the reaction, the solution acquired a purple-blue color. To stabilize and enhance sensitivity toward gelatinase, a series of mixed self-assembled monolayer (SAM)-protected GNDs@gelatin with different feed ratios of the two thiol-containing conjugate molecules (MCH and MPA) was prepared while keeping the total thiol concentration unchanged. Here, these GNDs@gelatin are referred to as GNDs@gelatin_X:Y_ (X:Y indicates the feed ratio of MCH to MPA). Taking GNDs@gelatin_1:49_ as an example, briefly, MCH (20 μL,10 mM) and MPA (980 μL,10 mM) were added to the GNDs@gelatin solution, and the mixture was incubated at 37 °C for another 1 h. The mixture was then centrifuged for 10 min at 10,000*g* to remove the excess gelatin, MCH, and MPA. To determine the sensitivity and stability of GNDs@gelatin with respect to MMP-2 and different pH values (PBST buffer; pH 5.5, 6.5, 7.0 and 7.5), the hydrodynamic sizes were measured in buffer solutions at different pH values. The stability of GNDs@gelatin in different conditions were monitored by UV–vis absorption. The absorption values were read from 400 to 800 nm wavelength in increments of 1 nm. All results were obtained by using 150 μL of solution in cuvettes, and the PBST buffer was used as the blank in all cases. For the MMP-2 response assay, 1.5 μg of activated MMP-2 was added into 500 μL of GNDs@gelatin, and the mixture was incubated at 37 °C for 6 h. All of the solutions were analyzed with a UV–vis absorption spectrophotometer, which recorded their spectral profiles after a 6 h of reaction time with MMPs.

### Characterizations

Transmission electron microscopy (TEM, H-7650, Hitachi, acceleration voltage = 120 kV) was applied to characterize the morphology of nanoparticles. UV–vis spectra were measured with a Beckman UV–vis spectrophotometer. The hydrodynamic sizes of nanoparticles were determined by DLS using a Malvern zetasizer (NanoZS, Malvern) with a 90° scattering angle at 25 °C.

### Cell Lines

MCF-7 breast cancer cell lines; CT-2A, U87-MG, and C6 glioma cell lines; MES-SA uterus cancer cell line; and A549 lung cancer cell line were obtained from the Food Industry Research and Development Institute (FIRDI, Hsinchu, Taiwan). All cell lines were screened and tested negative for mycoplasma contamination by PCR analysis. MCF-7, U87-MG, C6, and CT-2A were cultured in DMEM and supplemented with 10% FBS, 100 U·mL^–1^ penicillin, and 100 mg·mL^–1^ streptomycin at 37 °C in a fully humidified atmosphere of 5% CO_2_. In addition, MES-SA and A549 cells were cultured in McCoy’s 5A and RPMI medium, respectively.

### Calcein-AM/Propidium Iodide (PI) Double Staining

To further verify the PTT efficacy, 5 × 10^4^ C6 or A549 cells were seeded in 24-well plates, incubated, and cultured at 37 °C with 5% CO_2_ overnight. Afterward, the culture medium was removed and replaced with fresh 10% FBS medium containing 100 μg·mL^–1^ of GNDs@gelatin. After 24 h of incubation, the medium was washed and replaced by PBS buffer, and cells were illuminated for 5 min by either 808 or 1064 nm laser irradiation. Subsequently, the treated cells were stained with Calcein-AM and propidium iodide (PI) for 30 min. Then the stained cells were further rinsed three times with PBS buffer before the imaging of calcein-AM/PI double staining.

### Cellular Uptake Study

To compare the cellular uptake efficiency of GNDs@gelatin_X:Y_, both optical microscopy and TEM were used for analysis. For optical microscopy observation, C6 (approximately 1 × 10^5^ cells) was seeded in a 24-well culture dish. After the cells attached, media were replaced with fresh medium containing 100 μg·mL^–1^ of GNDs@gelatin_X:Y_ and incubated at 37 °C for 18 h. At the end of the incubation period, cells were washed with PBS two times to remove unbound GNDs@gelatin_X:Y_ and subsequently observed by an inverted optical microscope.

### Matrix Metalloproteinase 2/9 Activity Assay Using Zymography

Briefly, cells were lysed in RIPA buffer (20 mM Tris pH 7.4, 150 mM NaCl, 2 mM EDTA, 2 mM EGTA, 0.1% sodium deoxycholate, 1% Triton X-100, 0.1% SDS) containing protease inhibitors (Sigma-Aldrich). BCA assay (Pierce) was performed to measure the protein concentration, and equal amounts of protein were loaded on an SDS-PAGE (10%) gel containing 0.1 mg·mL^–1^ gelatin. After electrophoresis, the gel was washed twice for 30 min in zymogram renaturing buffer (2.5% Triton X-100) with gentle agitation at room temperature to remove SDS and then incubated at 37 °C for 48 h in reaction buffer (50 mM Tris-HCl pH 7.4, 200 mM NaCl, and 5 mM CaCl_2_). After staining with Coomassie brilliant blue, MMP activity was identified as clear zones against a blue background. The zymography gels were scanned using Adobe Photoshop software (Adobe Systems, Inc., CA), and densitometric quantification using ImageJ was performed.

### Calculation of the Photothermal Conversion Efficiency for GNDs@gelatin

To assess the photothermal conversion abilities of GNDs@gelatin, we followed procedures reported in the literature.^26^ The photothermal conversion efficiency (η) of GNDs@gelatin upon 808 nm and 1064 nm light irradiation was estimated using the following equation:
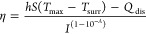
1where *S* represents the container’s surface area; *h* is the heat transfer coefficient; *T*_Max_ (unit: °C) and *T*_Surr_ (unit: °C) are the equilibrium temperature and ambient temperature of the surroundings, respectively; *Q*_dis_ corresponds to the heat dissipated from the light absorbed by the solvent and container; *I* (unit: mW) is the incident laser power; and *A* is the absorbance (OD = 1) of GNDs@gelatin at 808 and 1064 nm. The unknown parameter *hS* was evaluated using the following equations:
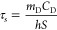
2
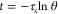
3
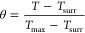
4where τ_s_ is the sample system time constant; mD and CD are the mass (1.0 g) and heat capacity (4.2 J·g^–1^) of the deionized water used as the solvent, respectively; θ is the dimensionless driving force temperature; *T* is a temperature for GNDs@gelatin solutions at a constant cooling time (*t*); and τ_s_ can be determined by applying linear time data from the cooling period vs −ln θ.

### Photostability of GNDs@gelatin

Both dispersed GNDs@gelatin and self-assembled GNDs@gelatin (100 μg·mL^–1^) were exposed to either 808 or 1064 nm diode continuous wavelength (CW) NIR laser irradiation (1 W·cm^–2^, 15 min, laser on). Subsequently, the NIR laser was turned off for 15 min, and the solution was naturally cooled to room temperature (laser off). The laser-on and -off cycles were repeated four times. A thermometer (TM-924C) from Lutron (Taipei, Taiwan) fitted with a K-type thermocouple (not exposed to the laser beam) was immersed in the GNDs@gelatin solutions to record the temperature. For control experiments, the same volume of water without the GNDs@gelatin solution was irradiated with CW laser, and its temperature was recorded. Meanwhile, the absorbance spectrum of the irradiated samples was examined after the last NIR irradiation.

### Mouse Tumor Model

Male nu/nu mice with body weights of 18–20 g purchased from BioLASCO Co., Ltd. (Yi-Lan, Taiwan) were housed under standard conditions (25 ± 2 °C/60% ± 10% relative humidity) with 12 h light/dark cycle. All animal procedures were performed in accordance with the Guidelines for the Care and Use of Laboratory Animals of National Health Research Institutes, Taiwan, and approved by the Institutional Animal Care (Taiwan) and Use Committee of National Health Research Institutes, Taiwan. Approximately six-week-old male Nu/Nu nude mice were anesthetized using isoflurane, and C6 cells were injected subcutaneously into the back-right flank (1 × 10^6^ cells). Tumors were grown until the mean volume had reached approximately 100 mm^3^. For *in vivo* PTT experiments, tumor-bearing mice were split into five groups on day 0, and each group included three mice: (1) control; (2) GNDs@gelatin only; (3) 808 nm laser only; (4) GNDs@gelatin +808 nm laser; and (5) GNDs@gelatin +1064 nm laser. Briefly, the mice in the control groups 1 and 3 received an intravenous injection with sterilized PBS solution (100 μL per mouse). The mice in groups 2, 4, and 5 were intravenously injected with 100 μL per mouse of 1 mg GNDs@gelatin in PBS solution. After 24 h, the mice in groups 2 and 4 were illuminated by 808 nm laser (1 W·cm^–2^, 10 min), and group 5 was illuminated by 1064 nm laser (1 W·cm^–2^, 10 min). After the above treatments, tumor volume and mouse weight were measured daily until day 26. Tumor volume was measured using a Vernier caliper and calculated as volume = (tumor length) × (tumor width)^2^/2. At the end of the experiment, all of the animals were euthanized. Animals were euthanized when the volume was over 3,000 mm^3^ or the weight loss was more than 15%, for ethical reasons. Tissues, including heart, liver, spleen, lung, kidney, and tumor, were harvested for histological examinations by H&E staining.

### *In Vitro* PA Signal Measurement of the GNDs@gelatin

For *in vitro* PA imaging, C6 cells were seeded in 6-well plate at a density of 5 × 10^5^ per well at 37 °C, 5% CO_2_. At 80% confluence, a series of concentrations of GNDs@gelatin from 10 to 300 μg·mL^–1^ were respectively added into the fresh culture medium and incubated for 24 h. The unlabeled C6 cells were considered as control. The cells were then permitted to recover in fresh PBS buffer for 1 h before being collected with 1X trypsin. During PA imaging, one milliliter (mL) of PBS solution containing 1 × 10^7^ GNDs@gelatin treated C6 cells was instantly added into 3% agarose gel model with holes. After setting, the samples were imaged by the PA system with the method mentioned above.

### In Vivo PAI of the Tumor

Male Nu/Nu nude mice aged 6 weeks were subcutaneously injected with C6 tumor cells (1 × 10^6^) in PBS suspension. Tumors were grown until the mean volume reached approximately 150–200 mm^3^. The tumor-bearing mice were anesthetized, and a layer of ultrasonic coupling gel was applied on each mouse’s skin at the tumor area. The tumor-bearing mice were monitored and imaged before and after intravenous injection of GNDs@gelatin (10 mg·mL^–1^, 100 μL per mouse, *n* = 3) using 808 and 1,160 nm lasers with the power intensity of 12 and 4 mJ·cm^–2^, respectively. At different postinjection times (at 0, 4, 24, and 48 h), PA images of tumor tissue were acquired and reconstructed.

### Gene Expression Array Analysis

RNA extractions from PTT-treated tumors (post-PTT 48 h) were isolated using RNeasy mini kit according to the manufacturer’s instructions. The 260/280 nm ratio was calculated using Nanodrop ND-1000. RNA integrity of each sample was confirmed by capillary electrophoresis resolving the 18S and 28S rRNA profile on the Agilent Technologies 2100 Bioanalyzer. Genome-wide microarray analyses were performed with 100 ng of total RNA using Affymetrix mouse gene 2.0 ST assay 2 chip. The differential expression genes (DEGs) were screened by mouse microarray expression profile and identified by the threshold: log2 fold change >5. To further achieve insight into the function of DEGs and genes in key modules, Gene Ontology, Kyoto Encyclopedia of Genes and Genomes pathway gene set (c2.cp.kegg.v7.2.symbols.gmt) was performed by clusterProfiler package and R version 4.0.2.

### Statistical Analysis

Measured values were expressed as mean ± standard deviation. Statistical significance was determined by two-tailed Student’s *t* test. The statistically significant difference was defined as *p < 0.05 and **p < 0.01.

## Results

**Figure 1:**
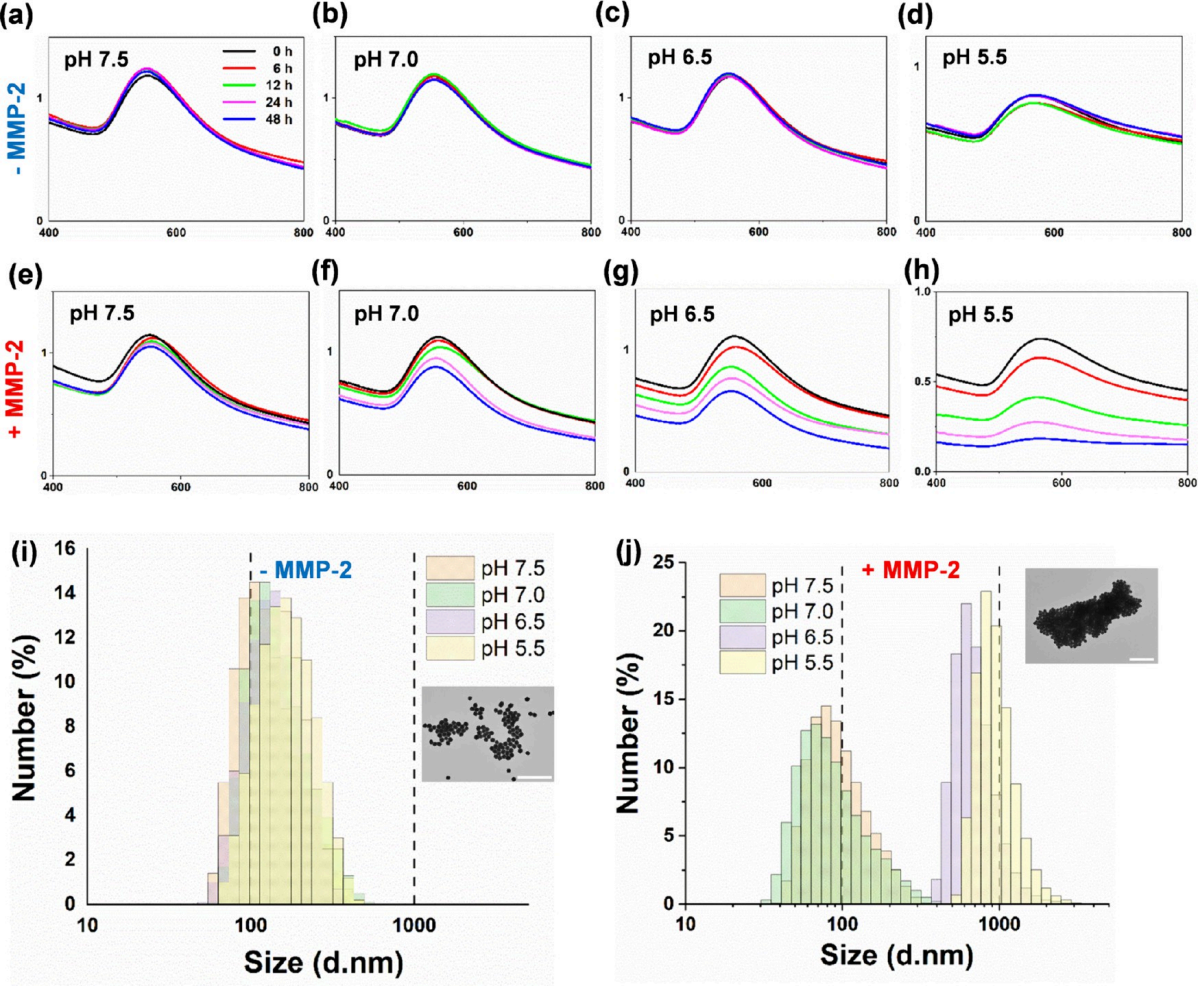
MMPs-triggered and pH enhanced self-assembly of GNDs@gelatin. (a–d) UV–vis–NIR spectra of untreated GNDs@gelatin at pH 7.5, 7.0, 6.5, and 5.5 after different incubation times. (e–h) UV–vis–NIR spectra of MMP-2 treated GNDs@gelatin at pH 7.5, 7.0, 6.5, and 5.5 after different incubation times. Time evolution of the GNDs@gelatin (i) before and (j) after MMP-2 treatments at different pH values of PBST solution (contain 500 ng·mL^–1^ MMP-2). Insets show TEM images of GNDs@gelatin after 6 h of incubation at pH 5.5. All scale bars are 5 μm.

To study the response of GNDs@gelatin to TME, GNDs@gelatin was incubated with activated MMP-2 in different pH-value PBST buffers. As shown in Figure 1, after 12 h of incubation, the hydrodynamic size of GNDs@gelatin at pH 7.5 and pH 7.0 conditions decreased from 120.4 ± 26.6 nm to 88.9 ± 4.5 nm, which mainly resulted from the degradation of GNDs@gelatin. It is worth noting that no discernible absorption change was observed at pH 7.5 and 7.0 conditions. Interestingly, GNDs@gelatin showed the greatest size increase to 750.9 ± 40.2 nm after incubation with MMP-2 for 12 h at low-pH conditions (i.e., pH 6.5 and 5.5), which was much larger than that of the initial ones. Further control experiments were conducted to validate the role of acidic environment in mediating MMP aggregation. We incubated GNDs@gelatin in pH 6.5 and 5.5 PBST buffer. As time passed, the hydrodynamic size of GNDs@gelatin increased slightly to 134.4 ± 22.6 and 152.3 ± 32.6 nm, respectively, which was largely due to the fact that the low-pH condition reduced electrostatic repulsion and shortened the interparticle distance between intact GNDs@gelatin.^27^ These results indicated that the self-assembly of GNDs@gelatin could not be triggered in the absence of MMPs. TEM images further demonstrated the observed self-assembly of GNDs@gelatin after 12 h of incubation. Our established GNDs@gelatin was revealed to be dual-triggered by the acidic pH and the upregulated MMP-2.-9 in the tumor microenvironment; it may be highly desirable for tumor-specific localization of nanotheranostics *in vivo*.

**Figure 2:**
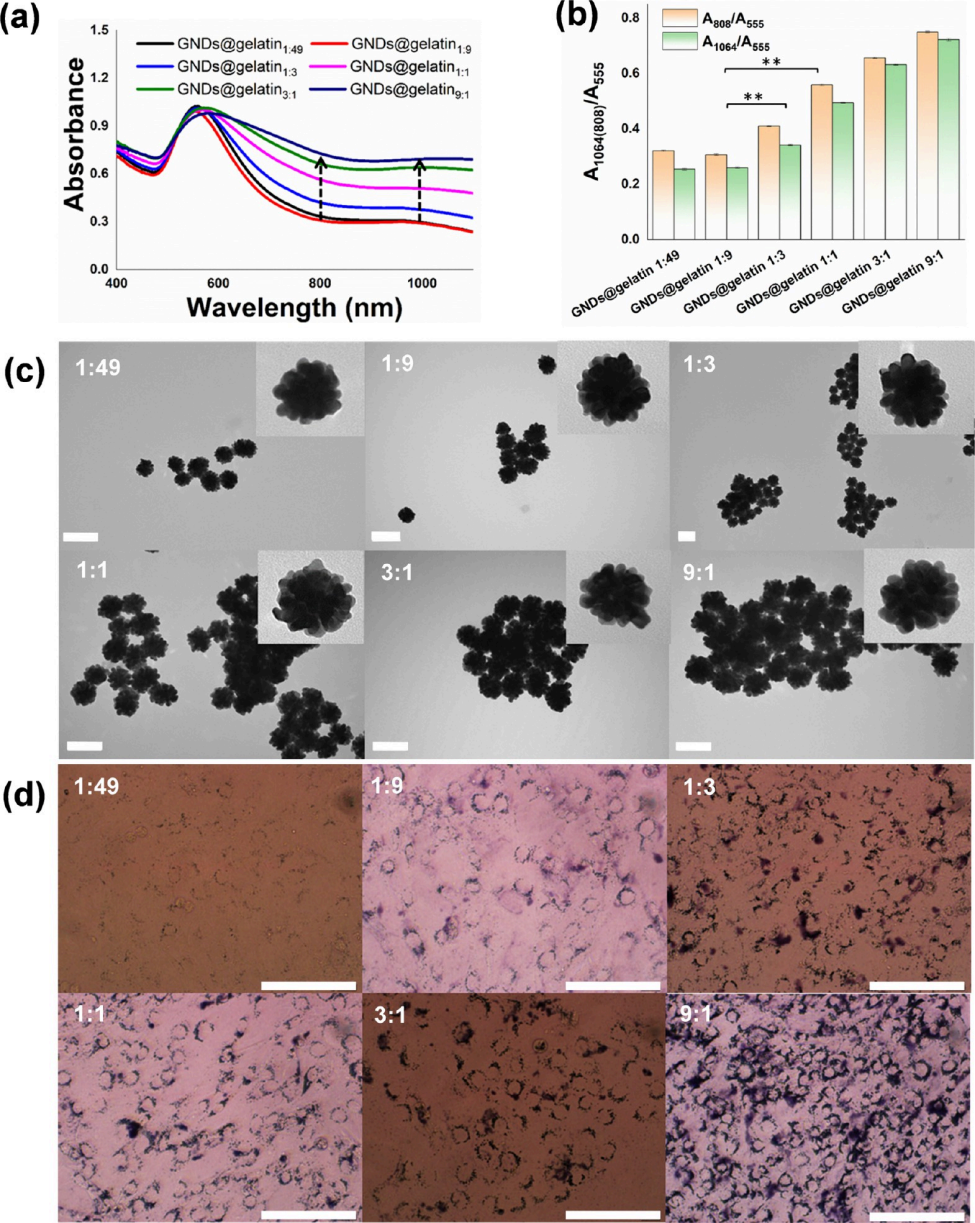
MCH-to-MPA ratio-dependent morphologies and absorption spectral evolution of GNDs@gelatin X:Y. (a) Spectral profiles of MCH:MPA (X:Y) ratio-dependent absorption of GNDs@gelatin X:Y in NIR I and II. (b) Variation of absorption ratios (A1064/A555 and A808/A555) versus the different feed ratios of MCH to MPA ligands that attached to the surface of GNDs@gelatin (**p < 0.01). (c) TEM images of GNDs@gelatin X:Y at different ratios of MCH to MPA. Insets show the structures of individual GNDs@gelatin X:Y under each condition. All scale bars are 100 nm. (d) Optical microscopy images delineate the uptake efficiencies of GNDs@gelatin X:Y by C6 cells at different MCH:MPA ratios. All scale bars are 100 μm.

Previous data showed that a ligand attached to the nanoparticle surface not only avoided biomolecule absorption on the nanoparticle but also increased attraction among the nanoparticles.^28^ To optimize and regulate the MMP response of GNDs@gelatin aggregation *in vitro*, different ratios of 6-mercaptohexan-1-ol (MCH) to 3-sulfanylpropanoic acid (MPA) were used to modify GNDs@gelatin and obtain various GNDs@gelatin_X:Y_. As shown in Figure 2a, when the feed molar ratio of MCH to MPA ligands ranged from 1:49 to 9:1, the modified GNDs@gelatin exhibited different UV–vis–NIR absorption spectra. As shown in Figure 2b, *A*_1064(808)_/*A*_555_ was employed to assess the modified GNDs@gelatin NIR absorption sensitivity. The dispersed GNDs@gelatins with MCH:MPA molar ratios below 1:9 showed relatively low absorption at the NIR region, while the other feed ratios above 1:9 failed to provide such a constant profile. Therefore, to effectively distinguish the “turn-on” PTT response upon TME-directed assembly of GNDs@gelatin, the molar ratio of 1:9 was selected for the subsequent *in vitro* and *in vivo* experiments. Interestingly, TEM images of GNDs@gelatin_X:Y_ revealed that the multibranched structure was retained in all of the different ratios (Figure 2c), and the results implicated that MCH and MPA modification might only affect the agglomeration state of GNDs@gelatin_X:Y_. As stated above, the TME-directed assembly of GNDs@gelatin via MMP enzymatic activity followed by cellular uptake dictates the efficiency of cancer photothermal therapy. Therefore, we assessed the efficiency by profiling the extra- and intracellular distributions of assembled GNDs@gelatin modified with different ratios of MCH to MPA. It can be seen from the bright field images that evident cellular uptake was observed for all of the ratios of GNDs@gelatin_X:Y_ in a ratio-dependent manner (Figure 2d and Figure S1).

The GNDs@gelatin_9:1_-treated group exhibited the highest cellular uptake amount of GNDs@gelatin, as observed from the large, black aggregates within the cells in the bright field image. It was also found that the bright field images of cells treated with GNDs@gelatin_1:49_ and GNDs@gelatin_1:9_ also showed cellular uptake of GNDs@gelatin and with less extracellular localization of aggregated GNDs@gelatin.

**Figure 3:**
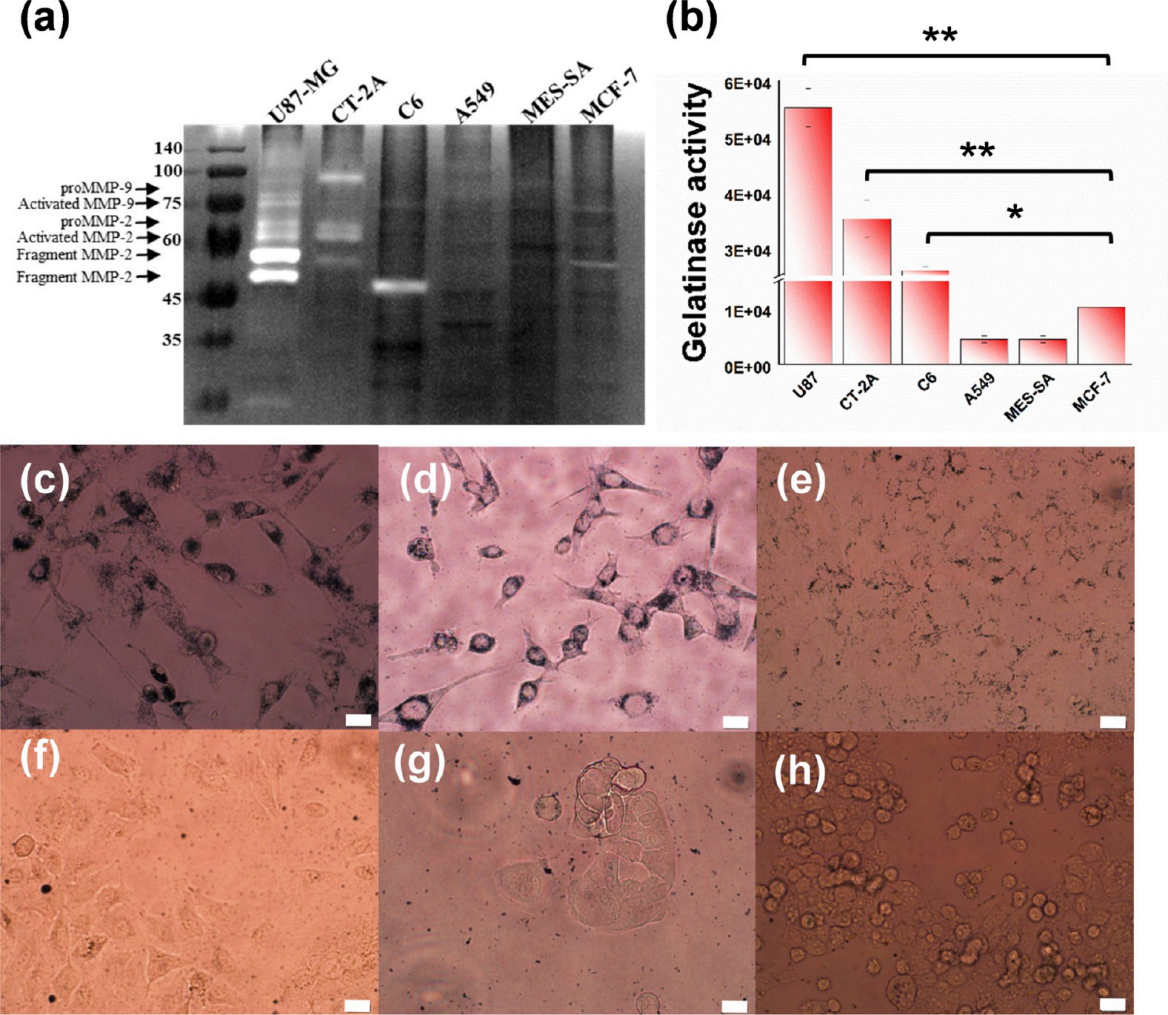
Effects of gelatinase activity on cellular uptake of GNDs@gelatin. (a) Representative analysis of cell lysate (20 μg of total protein per sample) subjected to SDS-PAGE followed by in-gel zymography. Molecular mass markers are indicated at the right side. (b) The densitometric quantification of MMP-2 and -9 activity. Differential optical microscopy images of (c) U87-MG, (d) CT-2A, (e) C6, (f) A549, (g) MES-SA, and (h) MCF-7 cells treated with GNDs@gelatin for 24 h. All scale bars are 20 μm (*p < 0.05 and **p < 0.01).

In our study, gelatin zymography was used to assess MMP activities by quantifying the intensity of both MMP-2 and MMP-9 activity bands on SDS-PAGE gels using densitometry. As shown in Figure 3a, several gelatinolytic activities were detected in all glioma cell lines (U87-MG, CT-2A, and C6), whereas it was faintly present in A549, MES-SA, and MCF-7cell lines. In each group, the proMMP-2, activated MMP-2, proMMP-9, and activated MMP-9 bands were scanned and quantified by densitometry in three independent experiments, and peak areas were averaged (Figure 3b). The activity of total gelatinase was at least 2.6-fold higher in glioma cell lines (*p* < 0.001) than in control groups. Furthermore, we employed an optical microscopy to visualize and locate GNDs@gelatin inside of the cells that express gelatinolytic activity (Figure 3c–h and Figure S2). Figure 3c–e shows GNDs@gelatin was located inside U87-MG, CT-2A, and C6 cells and clearly surrounded the nucleus. In contrast, Figure 3f–h shows a different distribution of GNDs@gelatin around A549, MES-SA, and MCF-7 cells; however, GNDs@gelatin inside of the cytoplasm was rarely found, which was likely due to the low expression of gelatinase compared to the above cells.

**Figure 4:**
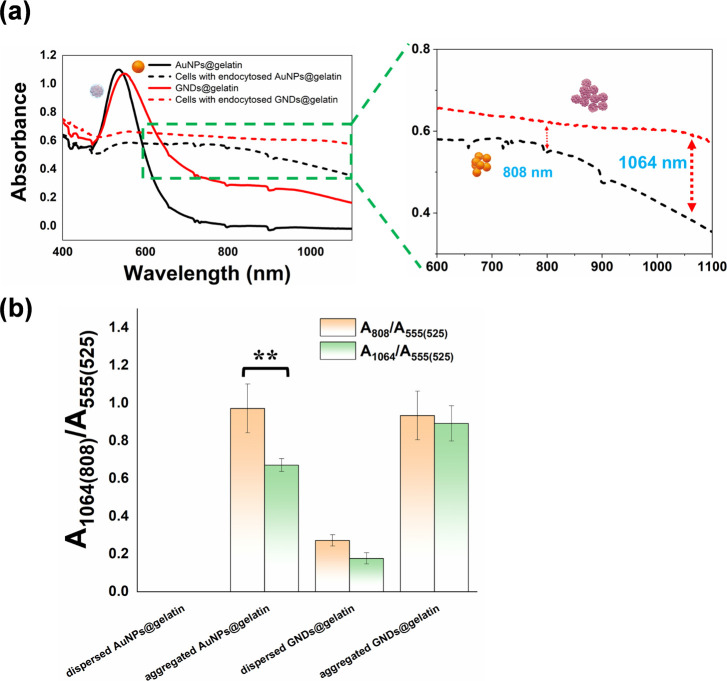
Activatable NIR plasmonic properties of GNDs@gelatin. (a) Normalized UV–vis–NIR absorption spectra of AuNPs@gelatin, GNDs@gelatin, endocytosed AuNPs@gelatin, and endocytosed GNDs@gelatin (solid black, solid red, dashed black, and dashed red lines, respectively). The inset shows the enlarged view of absorbance over the NIR-I and -II regions. (b) Comparison of *A*_1064(808)_/*A*_525_ of AuNPs@gelatin and *A*_1064(808)_/*A*_555_ of GNDs@gelatin before and after the nanoparticle endocytosis. Error bars were obtained from three experiments (**p < 0.01, *n* = 3).

Furthermore, in order to evaluate the extinction spectra of gold nanomaterials-treated cells, the CT-2A cells were incubated with the AuNPs@gelatin and GNDs@gelatin (200 μg·mL^–1^) for 24 h and then washed with PBS, trypsinized, centrifuged, and fixed with 2.5% glutaraldehyde to stabilize the spectra of endocytosed gold nanomaterials. As expected, the localized surface plasmon resonance (LSPR) peaks of both AuNPs@gelatin and GNDs@gelatin red-shifted and broadened in the NIR region after entering cells by endocytosis, which is a characteristic of the interparticle plasmonic coupling effect.^16,29^ An obvious decrease in the plasmon absorption at 525 nm (*A*_525_) and a strong increase in the surface plasmon band at NIR-I region were clearly discerned in the AuNPs@gelatin group. In addition, the overexpressed MMPs as gelatinase further induced the intracellular aggregation of GNDs@gelatin and resulted in the LSPR absorption band shifting to the NIR-I and NIR-II regions (Figure 4a). To quantify and analyze the aggregation degree of gold nanomaterials, the ratio of aggregated to dispersed gold nanomaterials was to be indicated by the ratio of the absorption value at either 808 or 1064 nm to that at 525 nm (AuNPs@gelatin) and 555 nm (GNDs@gelatin). It can be seen that the intracellular ratios of *A*_808_/*A*_525_ (AuNPs@gelatin) and *A*_808_/*A*_555_ (GNDs@gelatin) was 0.972 ± 0.002 and 0.936 ± 0.003 (*n* = 3), respectively. A substantial difference between AuNPs@gelatin and GNDs@gelatin was not found. Apart from the NIR-I region, the NIR-II absorption value at 1064 nm was employed to investigate the intracellular aggregation. As depicted in Figure 4b, comparing the absorbance ratio *A*_1064_/*A*_555_ (0.893 ± 0.003) to *A*_808_/*A*_555_ (0.934 ± 0.003), it remained almost unchanged for GNDs@gelatin. However, it is obvious that *A*_1064_/*A*_525_ (0.671 ± 0.002) decreased rapidly compared with *A*_808_/*A*_525_ (0.972 ± 0.002) for AuNPs@gelatin.

**Figure 5:**
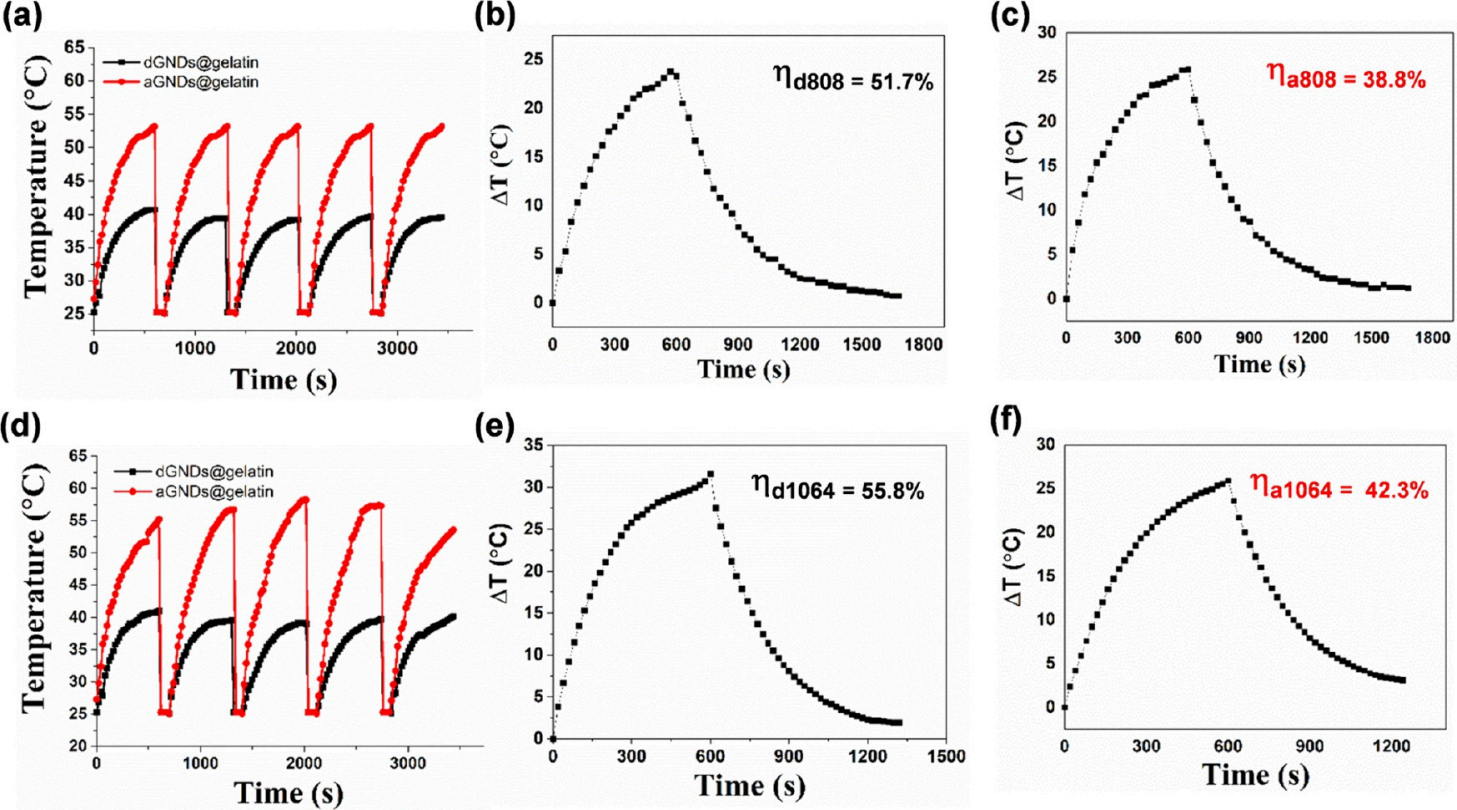
Photothermal properties of the synthesized GNDs@gelatin. Temperature elevation of dispersed and aggregated GNDs@gelatin over five laser on/off cycles of (a) 808 nm and (d) 1064 nm laser irradiation with the laser power density of 1.0 W·cm^–2^. The concentration of GNDs@gelatin was 100 μg·mL^–1^. The changes in temperature rise profiles were plotted as a function of the irradiation time for dispersed (b, e) (dGNDs@gelatin) and aggregated (aGNDs@gelatin) (c, f) GNDs@gelatin with different lasers. The photoirradiation was carried out using (b, c) 808 nm and (e, f) 1064 nm CW laser irradiation.

To further evaluate the potential of the synthesized GNDs@gelatin as a PTT/PA agent, photothermal stability and photothermal conversion efficiencies (η) were investigated. We irradiated GNDs@gelatin with either 808 or 1064 nm laser irradiation at 1.0 W·cm^–2^ for five continuous heating–cooling cycles (laser on/off) and monitored the solution temperature with irradiation time. The results showed that the GNDs@gelatin maintained the same temperature evolution profile during each irradiation cycle upon either 808 or 1064 nm laser irradiation, implying that the GNDs@gelatin possesses excellent photothermal stability (Figure 5a,d). Furthermore, the η values of GNDs@gelatin and its aggregates were examined. The temperatures of the aggregated GNDs@gelatin solutions rose quickly with time and reached 57 and 53 °C for 808 and 1064 nm laser irradiation, respectively, within 10 min, while the temperature of dispersed GNDs@gelatin showed no obvious change and leveled off at approximately 40 °C, suggesting that the aggregated GNDs@gelatin can rapidly absorb both NIR-I and -II light and efficiently convert the light energy into thermal energy. The η values, composed of temperature changes (Figure 5) and absorbance (eq 1), of the dispersed GNDs@gelatin were calculated as 51.7% at 808 nm and 55.8% at 1064 nm and decreased to 38.8% at 808 nm and 42.3% at 1064 nm as the effective radius increased (aggregated GNDs@gelatin).

**Figure 6:**
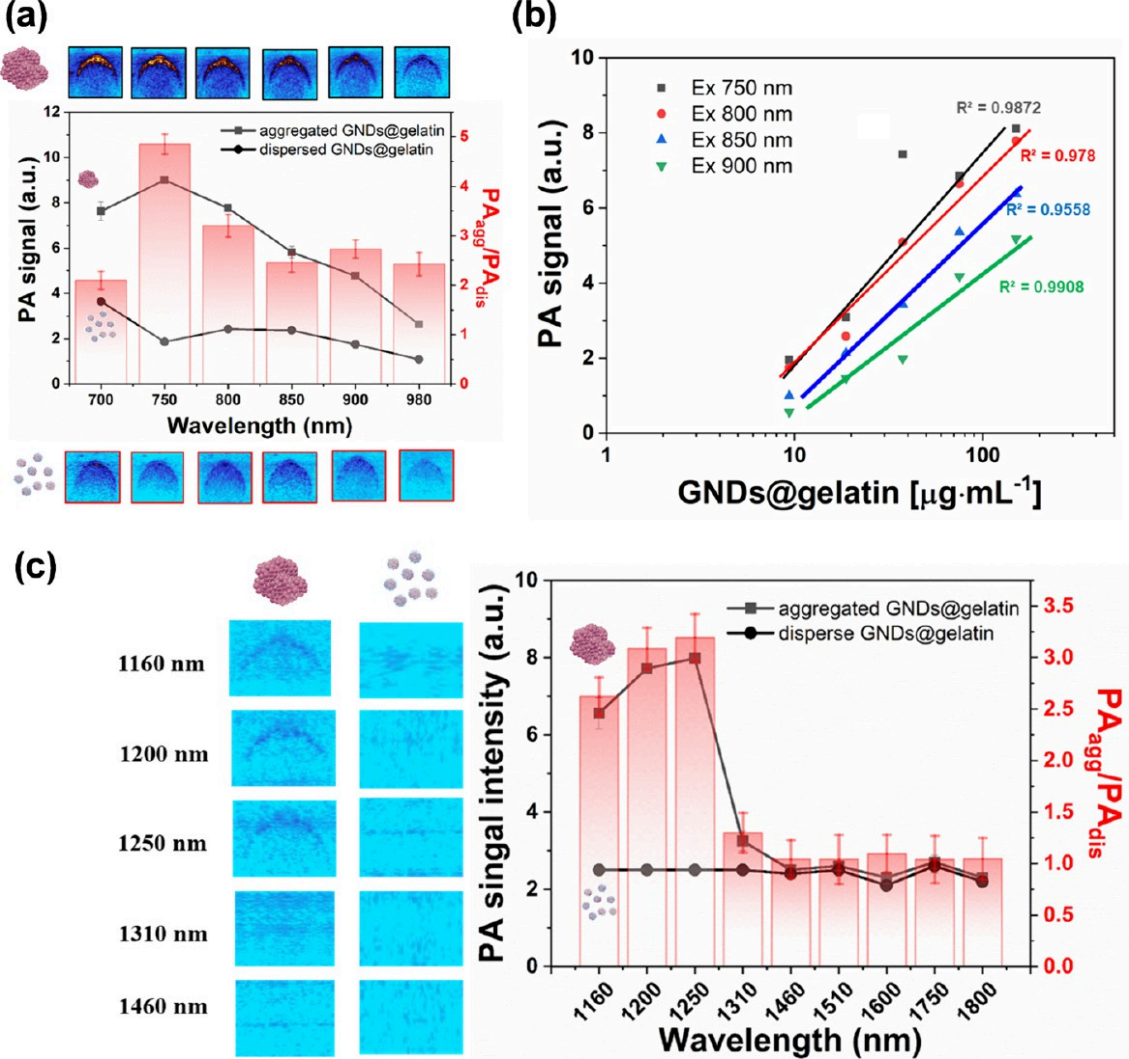
Photoacoustic characterization of the GNDs@gelatin. (a) Representative PA images and signal intensity of dispersed and aggregated GNDs@gelatin solutions at the concentration of 100 μg·mL^–1^, each was excited by the pulse laser from 700 to 980 nm. The corresponding B-scan images of aggregated GNDs@gelatin (top) and dispersed GNDs@gelatin (bottom) at different laser wavelengths are shown. (b) PA intensities of aggregated GNDs@gelatin as a function of nanoparticle concentration in PBS. R2 = 0.9872, 0.978, 0.9908, 0.9558 for 750, 800, 850, and 900 nm, respectively. (c) NIR-II photoacoustic imaging of GNDs@gelatin. Representative PA images (left) and derived signal intensities (right) of dispersed and aggregated GNDs@gelatin solutions with the concentration of 100 μg·mL^–1^ excited by pulsed laser at various wavelengths.

After confirming the excellent photothermal stability and photothermal conversion efficiencies, we next investigated the PA properties of the as-prepared GNDs@gelatin. Figure 6a shows the PA images and the corresponding PA spectra of both suspended and aggregated GNDs@gelatin at the same concentration (100 μg·mL^–1^) by pulsed laser irradiation ranging from 700 to 980 nm. Overall, the PA signals of aggregated GNDs@gelatin were stronger than those of dispersed ones. Specifically, the PA signal of aggregated GNDs@gelatin at 750 nm was 4.8-fold higher than that of dispersed GNDs@gelatin. Interestingly, we found that the average PA intensity (*Y*) was logarithmically proportional to aggregated GNDs@gelatin concentration (*X*) (*Y*_750_ = 2.3187ln(*X*_750_) – 3.3162, *R*^2^ = 0.9872; *Y*_800_ = 2.3295ln(*X*_800_) – 0.36705; *R*^2^ = 0.978, *Y*_850_ = 2.0178ln(*X*_850_) – 3.6537, *R*^2^ = 0.9908; *Y*_900_ = 1.7249ln(*X*_900_) – 3.5752, *R*^2^ = 0.9558) (Figure 6b). The good linear correlation between the PA signals and the concentrations of aggregated GNDs@gelatin was observed at different wavelength laser irradiation, indicating the feasibility of signal quantification. Moreover, the PA spectra in an aqueous solution were collected and normalized by a tunable pulse-laser illumination at the NIR-II region (Figure 6c). The PA signal intensity reached the highest value at approximately 1250 nm. In addition, the bright PA images indicated that aggregated GNDs@gelatin possesses excellent PA property, and the PA signal-to-noise ratio at 1250 nm was approximately 3.2-fold higher than that of dispersed GNDs@gelatin. The data explicitly demonstrate that MMP-responsive GNDs@gelatin is suitable for NIR-II PA imaging.

**Figure 7:**
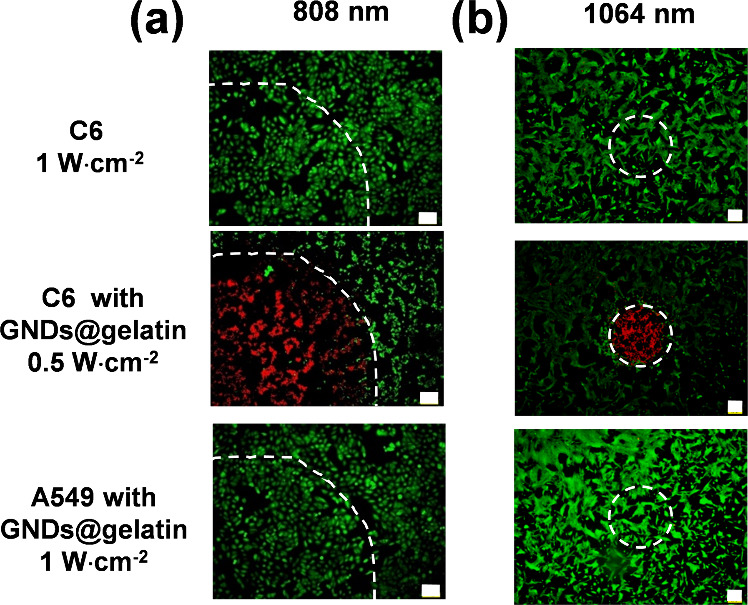
Photothermal cytotoxicity of GNDs@gelatin. (a) Fluorescence images of live (calcein AM, green) and dead (PI, red) C6 and A549 cells after treatment with 808 nm laser alone, or the GNDs@gelatin with 808 nm laser irradiation (scale bars are 100 μm). (b) Fluorescence images of C6 and A549 cells after incubation with GNDs@gelatin under the 1064 nm laser irradiation (scale bars are 200 μm).

To further elucidate the specificity and capability of NIR-I and -II photothermal tumor cell ablation by MMP-activated GNDs@gelatin, MMP-2 overexpressed C6 glioma cells and MMP-2 low expressed A549 cells were incubated with 100 μg·mL^–1^ of GNDs@gelatin for 24 h. After incubation, the culture medium was removed and then replenished with a fresh medium. As shown in Figure 7a,b, in the control groups treated with NIR laser irradiation alone, there was no obvious cytotoxicity. This suggests that both 808 and 1064 nm laser treatment had no obvious effect. This suggests that both 808 and 1064 nm laser treatment had no obvious effect on their cell viabilities. However, remarkable dead cell staining (99.9%) and pronounced low-viability were observed in the group treated with the GNDs@gelatin after the photoirradiation. A significant difference of cell viability was also found between GNDs@gelatin-treated C6 and A549 groups with combined either 808 or 1064 nm laser operations, indicating that such effective photothermal damage was specifically activated by gelatinase. According to the skin-tolerance threshold set by the America National Standards Institute, a 1064 nm laser has a higher maximum permissible exposure (MPE) of 1 W·cm^–2^ in comparison to that of an 808 nm laser (0.33 W·cm^–2^) (ANSI Z136.1-2007).^30^ Our established GNDs@gelatin can effectively kill cancer cells *in vitro* under the 1064 nm laser with a low power density (0.5 W·cm^–2^), indicating the strong potential application of clinical translation.

**Figure 8:**
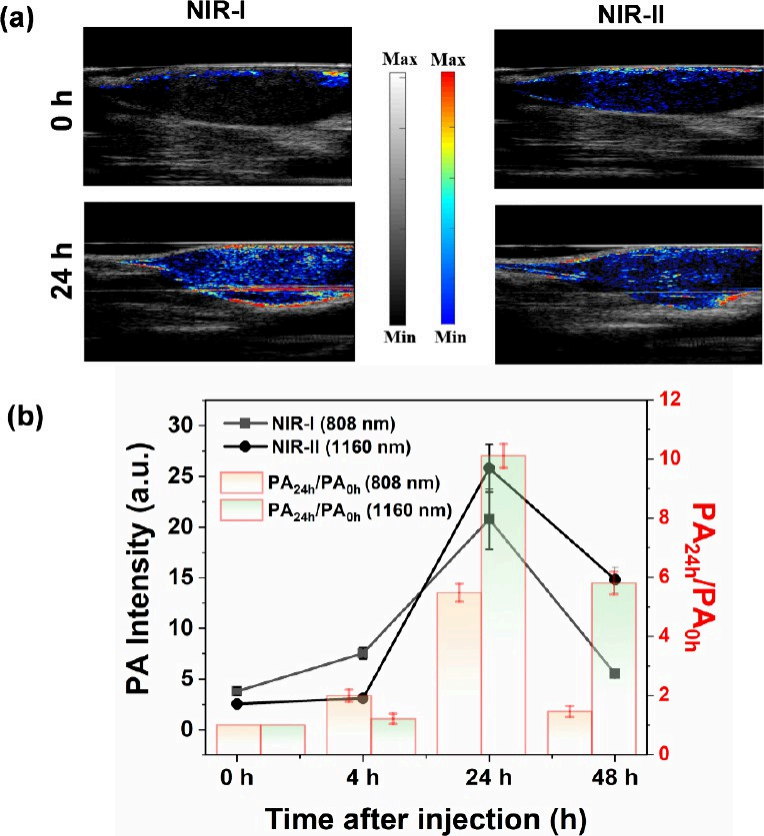
*In vivo* PA images of the GNDs@gelatin in the tumor tissues. (a) PA images at 808 nm (NIR-I) and 1160 nm (NIR-II) of GNDs@gelatin before (0 h) and after intravenous injection of GNDs@gelatin (24 h). (b) Time-dependent changes of PA intensities at 808 and 1160 nm following the GNDs@gelatin injection.

After systemic administration of GNDs@gelatin into the mice through the tail vein, the PA images were longitudinally recorded and quantified under the excitation of pulse laser at 800 nm. The PA intensities gradually increased for GNDs@gelatin-treated mice and reached their maxima at 24 h postinjection, which illustrates that 24 h after injection was determined to be the optimal time for PTT of tumor. At this time point, the PA intensity for GNDs@gelatin-treated mice was 5.5-fold higher than that of preinjection of GNDs@gelatin, indicating that the GNDs@gelatin can serve as a long-term PA contrast agent *in vivo* (Figure 8a,b). Encouraged by the aforementioned excellent *in vitro* and *in vivo* responsive NIR-I PA imaging results, we further studied NIR-II PA imaging of the GNDs@gelatin *in vivo*.

**Figure 9:**
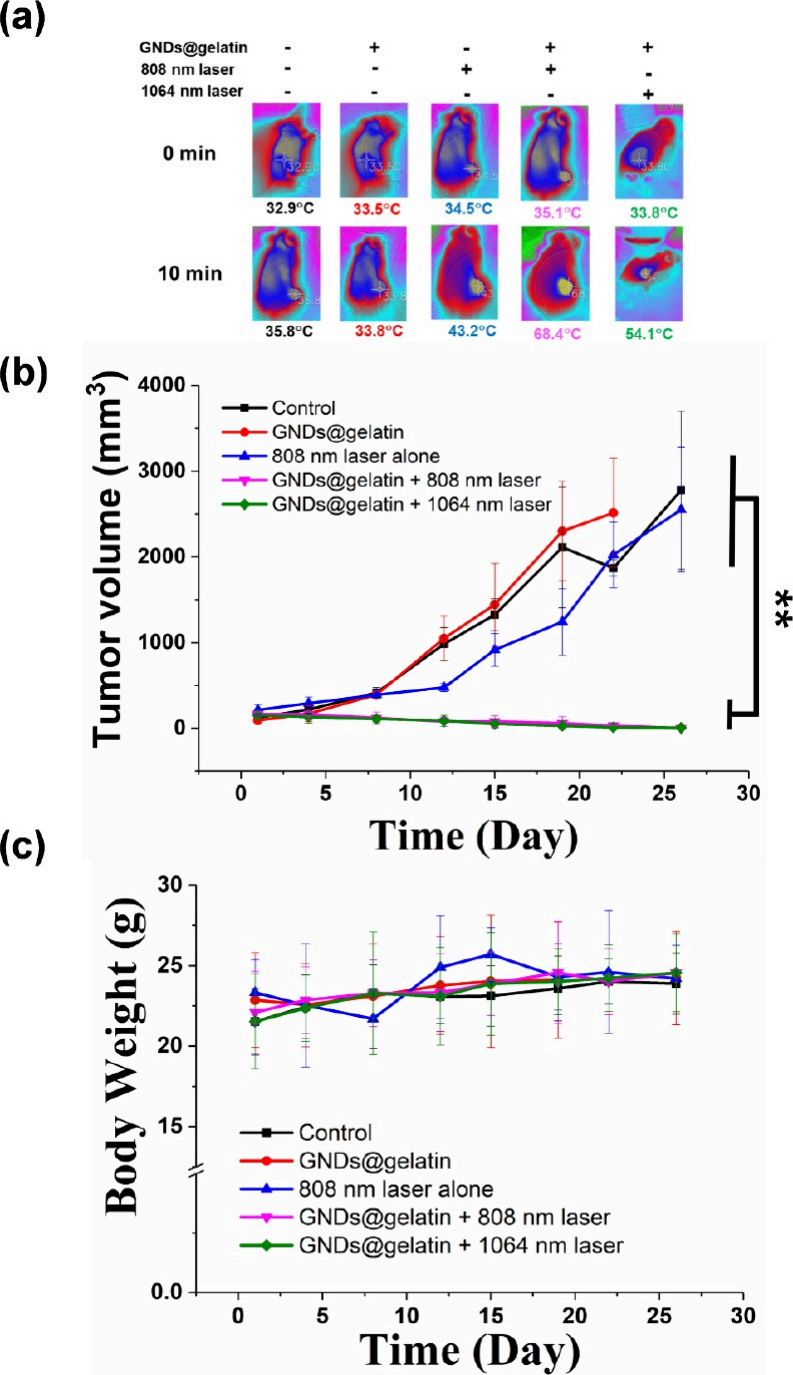
*In vivo* NIR-I and -II PTT against tumor. (a) Representative IR thermal images of C6-tumor-bearing mice with intravenous injection of GNDs@gelatin under laser irradiation at either 808 nm (1 W·cm^–2^) or 1064 nm (1 W·cm^–2^) for different time periods (**p < 0.01, *n* = 5). (b) Tumor growth curves with different treatments over the period of 30 days. The significant inhibition of tumor growth was observed under the NIR-I or NIR-II operation in the presence of GNDs@gelatin. (c) The corresponding body weight variation of mice following the treatments (**p < 0.01, *n* = 5).

In light of the *in vitro* PTT results, the photothermal therapeutic effect of the gelatinase-responsive GNDs@gelatin was further investigated *in vivo*. The maximum NIR PA signals in the tumor region could be detected at 24 h postinjection. PTT was conducted accordingly with an 808 or 1064 nm laser irradiation (1 W·cm^–2^) for 10 min, and the heating process was monitored every minute by an infrared (IR) camera. As shown in Figure 9a, after 10 min of irradiation of either 808 or 1064 nm laser, the tumor local temperature was raised by 33.3 and 20.3 °C, respectively. On the other hand, the local temperature of the control tumor was only increased by 8.7 °C upon the same dose of 808 nm laser irradiation if no GNDs@gelatin administration was applied. To further evaluate the antitumor effect for the NIR-I and NIR-II PTT using GNDs@gelatin, tumor volumes were continuously monitored for 4 weeks. As indicated in Figure 9b, the growth of tumors for GNDs@gelatin treated mice was significantly suppressed without reoccurrence observed after both 808 and 1064 nm photoirradiation. This might be due to the ability of GNDs@gelatin to self-assemble in the presence of MMPs, which leads to the elevated photothermal property. In contrast, saline-treated mice with 808 nm laser irradiation failed to show any antitumor capability. In addition, no notable therapeutic effect was identified for both GNDs@gelatin- and saline-treated mice without NIR laser irradiation. These results showed that *in situ* assembly of GNDs@gelatin offers excellent activatable photothermal therapeutic ability against tumors. Moreover, there was no obvious body weight loss observed for all of the mice throughout the experimental period (Figure 9c).

**Figure 10:**
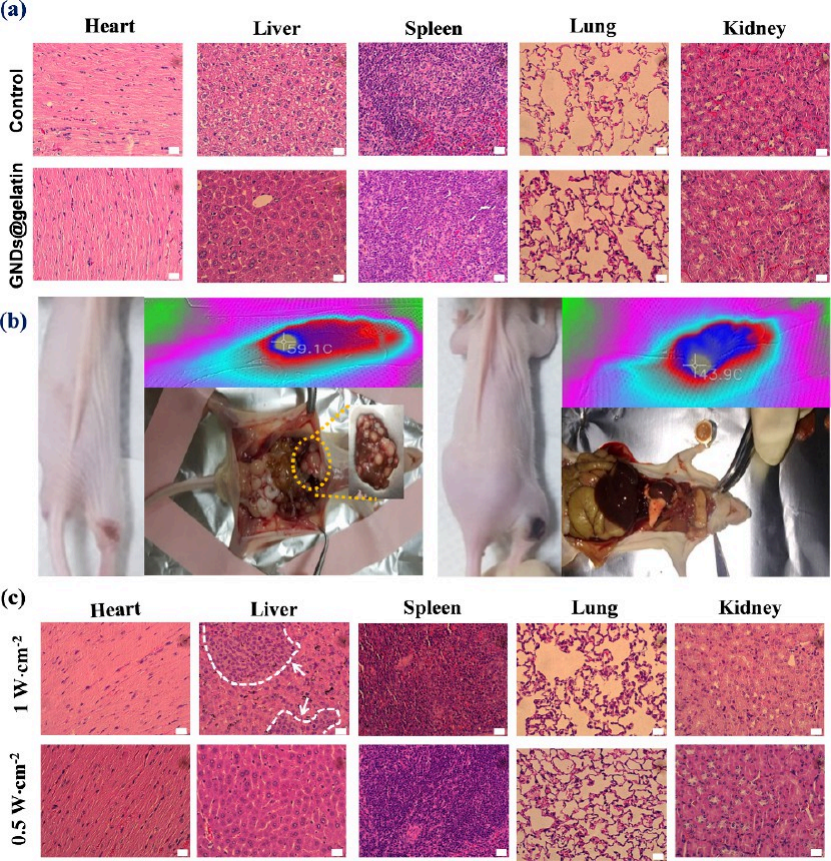
Modulation effect of laser power on tumor metastasis following PTT. (a) Representative H&E-stained images on histological sections of major organs from mice 7 days post i.v. injection of GNDs@gelatin (1 mg per mouse) and laser irradiation (NIR-I at 808 nm). (b) IR images and representative pictures of tumor bearing mice at 28 days after 1.0 W·cm^–2^ (left, 60 °C average final temperature) and 0.5 W·cm^–2^ (right, 44 °C average final temperature) NIR-II (1064 nm) PTT treatments. GNDs@gelatin-injected mice with complete tumor eradication were sacrificed 28 days after NIR-II PTT treatment. The liver metastasis of tumor was observed under the 60 °C average final temperature using higher laser power at 1.0 W·cm^–2^ (shown with dashed yellow circle and enlarged inset in the left panel), whereas no metastasis was induced under the 44 °C average final temperature using 0.5 W·cm^–2^ (right panel). (c) H&E-stained images of major organs from the mice in panel b. The distinct traits of liver metastasis (indicated by white arrow and white dashed lines) of C6 glioma tumor were verified from the mouse group treated with the 1.0 W·cm^–2^ laser. All scale bars are 20 μm.

Light with a spectral range in the NIR-II window with deeper tissue penetration and higher maximum permissible exposure compared to traditional NIR has gained popularity as a potent tool for noninvasive phototherapy. The histological assessment further confirmed the absence of pathological damage in the major organs such as heart, liver, spleen, lung, and kidney 28 days after PTT treatment (GNDs@gelatin/1064 nm group). As shown in Figure 10a, there were no significant histological differences between untreated control mice and mice treated with GNDs@gelatin, suggesting the excellent biocompatibility of GNDs@gelatin associated with photothermal therapy. More interestingly, in NIR-II PTT, it is observed that GNDs@gelatin mediated-PTT conforms to the conventional “more is better” paradigm, wherein the greater power density of the 1064 nm laser generates higher cell/local heating and thereby more cell death. As shown in Figure 10b, scar formation at the laser irradiation site occurred, and the old skin later peeled off, followed by new skin formation while the mice were exposed to 1064 nm with a power density of 1 W·cm^–2^ and the average final temperature reached 60 °C. Unexpectedly, yet importantly, we found that higher power density of PTT potentiated the growth of distant liver metastases at 28 days post-PTT treatment. At the end of the study, mice were sacrificed and assessed for the extent of metastasis to the major organs by H&E staining. Copious tumor nodules were found on the liver of those receiving PTT with higher power density (2/3), compared to no tumor nodules observed in the mice exposed to 1064 nm with a power density of 0.5 W·cm^–2^, and the average final temperature reached 45 °C for the group (3/3) (Figure 10c).

**Figure 11:**
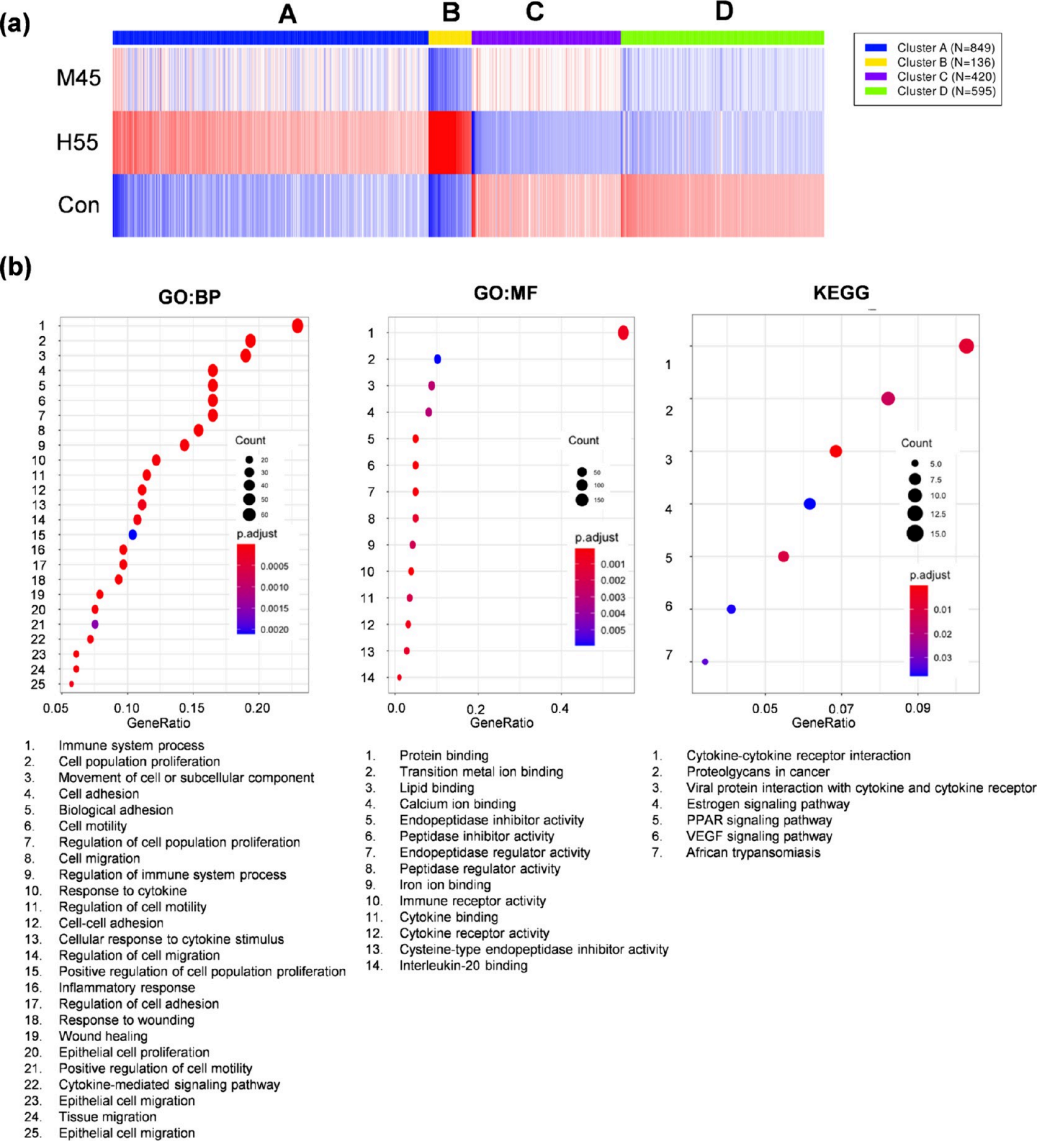
GNDs@gelatin PTT induced differentially expressed genes (DEGs) in variant condition. (a) Heatmap of intersample correlation showed there was an obvious difference of significant mRNA expression levels between GNDs@gelatin PTT-treated and untreated groups. The Pearson’s correlation coefficient is represented by a color scale. The intensity increased from blue (relatively lower correlation) to red (relatively higher correlation). Correlation was evaluated by Pearson’s correlation coefficient of significant mRNA expression levels. (b) Bulb map of GO:BP, GO:MF, and KEGG analysis of differentially expressed gene. Rich factor represents the enrichment degree of differentially expressed genes. The Y axis shows the name of enriched pathways. The area of each node represents the number of the DEGs. The *p*-value is represented by a color scale. The statistical significance increased from purple (relatively lower significance) to red (relatively higher significance.

To further elucidate the underlying mechanism of liver metastasis caused by high-temperature PTT, genome-wide microarray analyses were performed to compare gene expression between the high-temperature PTT (H55) group and the mild-temperature PTT (M45) one. By comparing the transcriptome counts of the various genes and subsequently applying the cutoff criteria, 382 genes were identified as DEGs. Subsequently, a heatmap of DEGs was created; the mRNA expression profiles of H55, M45, and untreated tumor resulted in obviously separate clusters (Figure 11a, Table S1). To gain further insight into the function of identified DEGs for metastasis, gene enrichment analysis was performed using gProflier2 package,^31^ including Gene Ontology and KEGG pathway enrichment analyses (Figure 11b). GO enrichment analysis showed that DEGs were significantly enriched in 179 biological processes (BPs) and 21 molecule functions (MFs). Furthermore, KEGG pathway enrichment analysis indicated that DEGs were significantly enriched in 7 pathways (Figure S3 and Table S2).

## Discussion

Conventional nanotheranostic formulations for cancer suffer from undesirable off-targeting properties, such as intrinsic nonspecific pseudosignal with high background signals and low tumor-to-normal tissue signal ratio, and their ability to destroy healthy tissues. To adequately address these disadvantages and achieve precision therapy, GNDs@gelatin has been rationally designed to overcome these limitations; the always-on pattern relies on the endogenous stimuli of the tumor microenvironment. Our previous report revealed that gelatin grafted on the surface of GNDs can not only help to shield GNDs in blood circulation and protect GNDs@gelatin from unspecific uptakes, but also enable GNDs@gelatin to target and accumulate in gelatinase-overexpressing cells.^32^ In this study, our GNDs@gelatin accomplished to improve therapeutic efficacy by successively increasing intracellular accumulation and shifting its absorption band into the NIR-I and -II regions.

MMPs are first expressed as latent enzymes (proMMPs) and can be activated by action of a membrane-type MMP in the pericellular and extracellular compartments.^33^ In the tumor microenvironment, GNDs@gelatin will be digested by the activated gelatinase and reduced to small-sized ones, which are internalized by tumor cells followed by their subsequent assembly at cytoplasm. Previous data revealed that MCH modification on AuNPs-gelatin can accelerate the modified AuNPs to aggregate after MMPs digest the gelatin on the AuNPs-gelatin, which is adequate for the application of targeting ability toward the MMP-2/-9 overexpressed tumors. It is thought that MCH served not only to block the surface space to avoid residue of digested gelatin absorption on the AuNPs; the terminal −OH group also increase the attraction among the AuNPs.^28^ That is, MCH modification might affect GNDs@gelatin colloidal dispersity/stability. Therefore, the loss of stabilizing gelatin molecules from the surface can facilitate aggregation of GNDs@gelatin. It was found that the GNDs@gelatin modified with an MCH:MPA molar ratio above 1:3 showed a broadband absorption ranging from the NIR-I to NIR-II window, whereas GNDs@gelatin_1:49_ and GNDs@gelatin_1:9_ exhibited a narrow LSPR centered at 555 nm. It should be emphasized that the distinct difference in absorption at the NIR region before and after MMP-directed assembly is the key factor for GNDs@gelatin to successfully display a “turn-on” PTT response. If the absorption bands of dispersed GNDs@gelatin at the NIR region are too strong and broad, e.g., GNDs@gelatin_9:1_, it is difficult to discern the “turn-on” PTT response potentiated from the assembly-induced enhancement of absorption, and therefore, the imaging sensitivity and specificity are markedly diminished. Considering collectively both the NIR-absorption contrast before and after assembly, and cell-uptake efficiency of various GNDs@gelatin_X:Y_, we selected GNDs@gelatin_1:9_ for our following *in vitro* and *in vivo* “turn-on” PTT studies. GNDs@gelatin undergoes digestion-induced self-assembly, and consequently PA and PTT properties are elevated, which offers tumor-targeted imaging and therapy. It should be emphasized that the kinetics of aggregation is the key factor influencing the cellular uptake behavior of GNDs@gelatin. If the aggregation is too fast, i.e., earlier than taken up by cancer cells, a fraction of nanoparticles would remain in the surrounding tissue environment, which results in nonspecific heating and their ablation. Augmented intracellular localization and assembly of GNDs@gelatin are important to shift its plasmonic absorption band to the NIR region. Such intelligent “turn-on” nanomaterials circumvent the limitations of “always-on” nanomaterials, which are the intrinsic nonspecific pseudosignal with high background signals and low tumor-to-normal tissue signal ratio.

It has been reported that the activity of MMPs was significantly elevated in malignant gliomas, compared to that in low-grade glioma and normal brain tissues.^21,34^ MMPs make strong candidates for targets for systemic delivery for controlled release of chemotherapeutics, unlike many biomarkers that show significant differences in expression between primary and metastatic tumors. Very recently, Yang et al. reported MMP-induced assembly of gold nanoparticles for imaging-guided PTT in the NIR-I region.^35^ Thus far, however, TME-triggered assembly of AuNPs has rarely been used for PTT in the NIR-II region. The reason for this is ascribed to the absorbance of their intracellular aggregates being poorly overlapped with the excitation laser NIR-II wavelength. Unlike AuNPs-based systems, we further demonstrated that aggregated GNDs@gelatin retained its absorbing capabilities in the NIR-II region, which makes it a strong potential candidate for clinical translation.

Reabsorption has been suggested as an explanation for high PCE in multibranched nanostructures. The η value is determined by the fraction of the absorption in the extinction (the sum of the absorption and scattering). Wang et al. used a finite difference time-domain (FDTD) method to calculate the theoretical η of 8 Au nanocrystals. They observed the theoretical η values were not in accordance with the experimental results when the particle radius was above ∼15 nm. The observed difference between the experiments and calculations can be ascribed to the reabsorption of the scattered light by the nearby nanocrystals.^36^ Reabsorption has been suggested as an explanation for similar observations in other nanoparticle systems, such as gold-nanorod-decorated TiO_2_ rambutan-like microspheres.^37^ The multibranched nanostructures greatly increase the chance of reflected photons from all directions to revisit the surface and thereby stimulate the reabsorption of the reflected photons on the surface of nanostructures.

It was noted AuNP assembly and increasing size would increase LSPR scattering, resulting in a significant decrease in η.^26,36^ The η value of aggregated GNDs@gelatin is markedly higher than those for gold nanorods (22%), gold nanoshells (13%), gold vesicles (18%), and gold nanovesicles (37%) that were previously reported in the literature.^38,39^ This importantly indicates the high efficiency of aggregated GNDs@gelatin in the conversion of NIR laser energy into heat due to the critical fact that the presence of the multibranched nanostructure could efficiently reabsorb scattered light.^9,11^

The photoacoustic signals from the endocytosed GNDs@gelatin *in vitro* were quantitatively analyzed. The PA spectra were collected and normalized by a tunable pulse-laser illumination from 700 to 1,800 nm (Figure 6a,c). The data suggested that endocytosed GNDs@gelatin is suitable for NIR-I and NIR-II PA imaging due to the excellent photon-caustic converting efficacy. Furthermore, the PA amplitudes of GNDs@gelatin at 750, 800, 850, and 900 nm were determined at a series of concentrations from 10 to 150 μg·mL^–1^ (Figure 6b), and all wavelengths displayed a linear relationship between PA signal and concentration. Encouraged by the *in vitro* PA imaging results, we further chose 808 and 1160 nm to evaluate the *in vivo* PA imaging. The PA intensity of GNDs@gelatin was related to the time post injection, which reached a maximal value at 24 h post injection at both 808 and 1160 nm (Figure 8). The PA intensity values of 808 and 1160 nm at 24 h postinjection were approximately 5.5- and 10.1-fold stronger than those of preinjection ones, respectively. Due to the limited power density of the 1064 nm pulse laser (40 mJ pulse peak energy at 1160 nm vs 80 mJ pulse peak energy at 800 nm), it could be observed that the PA signals of the nanoprobe at 1160 nm were weaker than those at 800 nm at 24 h postinjection. However, a highly significant difference of PA intensity was observed between 24 h postinjection and preinjection mice. A maximum signal-to-noise ratio of 10.1 at 24 h postinjection was found, indicating the promising PA contrast of GNDs@gelatin and its superior feature of NIR-II PA imaging.

In light of the tumor PA imaging results, the PTT capability and efficacy were investigated in the subcutaneous xenograft C6 mouse tumor model. As maintained above, the maximum PA signals in the tumor region could be detected at 24 h after GNDs@gelatin injection. PTT was conducted accordingly with either 808 nm (1 W·cm^–2^) or 1064 nm (1 W·cm^–2^) laser irradiation for 10 min, and the heating process was monitored by an IR camera. The temperature of the GNDs@gelatin-injected tumor exposed to 808 nm laser increased rapidly from 35.1 °C to more than 68.4 °C in 10 min, while the maximum temperature of the saline injected ones was around 35.8 °C. In contrast, mice irradiated with a 1064 nm laser showed marginal temperature elevation in both groups. The tumor temperature of GNDs@gelatin-treated tumors that were exposed to 1064 nm increased and reached ∼54.1 °C. Considering the threshold temperature (43 °C) of PTT, these temperature changes generated by MMP-responsive GNDs@gelatin was high enough to induce tumor ablation. As indicated in Figures 9a and 10b, the growth of tumors for GNDs@gelatin-treated mice was significantly suppressed without reoccurrence observed after 1064 nm irradiation at both 1.0 and 0.5 W. Surprisingly, a higher PTT temperature potentiates metastasis and greatly accelerated the growth of metastatic liver tumors, while lower PTT temperature exhibited no metastatic liver tumors. Consequently, the GNDs@gelatin-treated mice with the lower PTT temperature showed better long-term survival than those with the higher temperature. Bear et al. conducted an elaborate study to evaluate the antitumor effect of gold nanoshell mediated PTT, revealing that PTT promoted the infiltration ability of myeloid-derived suppressor cells and enhanced the growth of distant lung metastases.^40^ Paholak et al., who compared 1.0 and 0.5 W PTT, implicated that higher temperature induced by 1.0 W PTT enhanced secondary tumor growth compared to 0.5 W PTT.^41^ However, the cause of the unexpected correlation between laser power density and higher incidence of liver metastasis remains unclear. In our study, we determined that maximum long-term survival and optimum tumor inhibition associated with minimum metastatic potential occurred with GNDs@gelatin-treated glioma cells with a laser power of 0.5 W·cm^–2^. So far, accumulated evidence suggests that the presence of optimal temperature which can enhance immune response by photothermal effects.^42−45^ Although PTT has been previously shown to prime immunogenic cell death for antitumor immunity, evidence that PTT can prime metastasis has been limited. In our study, we performed whole genome RNA-sequencing on tumors exposed to various treatments during the acute phase of thermal ablation (post-PTT 48 h) to determine the possible metastatic mechanisms involved. Among them, 315 DEGs were found between the H55 group and M45 group. Comparison with M45 group revealed that 237 genes were upregulated and 78 genes were downregulated in the H55 group. To study the function between H55 and M45 of GND photothermal treatment and the participated pathways, GO and KEGG enrichment analysis were performed for differentially expressed genes from in PTT-treated tumors. The roles of molecular mechanisms were investigated by GO/KEGG analysis by all gene sets, such as BP, CC, MF, and KEGG. The selected pathways that showed significant differences, ranked by −log_10_ (P value), are displayed in Figure 11. Top terms for photothermal treatment included positive regulation of immune responses, cell adhesion, cell proliferation, and cell migration. As shown in Figure S5, 54 genes associated with cell proliferation and 43 genes associated with cell migration were significantly regulated, respectively, upon H55 GND photothermal treatment, relative to M45 photothermal treatment by the criteria, |log_2_Fold Change| ≥ 6.

Furthermore, we compared the mean expression of two interested groups and found several core EMT genes, such as ERBB3, ZEB2, CDH2, DKK1, LOXL2, MGP, S100A14, and LSR. We found the gene expression fold change of ERBB3, ZEB2, DKK1, S100A14, FOXC1, and LSR is 3.23, −2.03, 2.63, 56.51, 3.11, and 3.39, respectively (Figure S4a). Based on the criteria of |log_2_ (fold change)| > 2, the result revealed the high-temperature H55 group induced significant differential expression of EMT-related genes group compared with the mild temperature M45 group. Moreover, integrin and chemokine genes, such as ITGA3 (7.39 fold), ITGB4 (11.15 fold), CXCL3 (6.48 fold), and CXCL2 (7.96 fold), showed significant upregulation to promote cell migration (Figure S5b). MMP in general have been implicated in many steps of malignancy, invasion, and metastatic progression. Expression MMPs has been directly associated with the EMT changes in various of cancers. The difference mRNA expression of MMP-1 (3.35 fold), MMP-3 (4.96 fold), MMP-9 (11.8 fold), and MMP-13 (9.46 fold) between H55 and M45 group can be considered as a clear EMT step (Figure S5c). In addition to EMT, the expressions of stemness-related markers are thought to drive resistance to therapy. We observed the stemness-related gene expression between these two interested groups and found the gene expression fold change of KLF4 and EpCAM is 3.34 and 6.3, respectively (Figure S5d). The results implied that the difference of therapeutic temperature may drive tumor relapse and therapeutic resistance.

From Figure S6a, it could be clearly seen that the expression of EMT related gene ZEB1 increased as the photothermal temperature increased from 45 to 55 °C with a relative fold change of 1.4, which indicates the onset of EMT at higher temperature. Then we quantified the expression of stemness related genes, such as Myc, Nanog, and GLS (Figure S6b–d). Irrespective of these genes, their expression levels were amplified at higher temperature; the relative fold changes from M45 to H55 were 3.3, 2.98, and 1.46 respectively. These results suggest that higher photothermal temperature increased the expression levels of EMT/stemness related genes, which could drive metastasis.

Overall, our results demonstrated that PTT-induced high-temperature hyperthermia modulated the disease progression in tumor metastasis and recurrence. In the future, we will apply the quantitative reverse transcription PCR (RT-qPCR) to further validate those DEGs closely associate with cancer metastasis and stemness profiled via microarray analysis. For example, in our preliminary study (data not shown), the high-temperature hyperthermia resulted from GNDs@gelatin-mediated PTT significantly induced epithelial-mesenchymal transition (EMT)-related gene expressions, such as twist, snail, slug, zeb1, and E-cad, implicating the potential to initiate the following cancer invasion and metastasis. Besides, such induced high-temperature hyperthermia could escalate the gene expressions of c-Myc, Sox-2, and Oct4, which are cancer stemness-related genes that play crucial roles in the upstream cascade of tumor growth, metastasis, and drug resistance.

## Conclusion

Taken together, these results suggest that combining GNDs@gelatin-mediated PTT with an optimal temperature window (50 °C > *T* > 43 °C) could improve long-term survival in animal models of glioma cancer and provide a viable treatment option for those with currently incurable metastatic disease. Of course, a successful cancer treatment must achieve effective therapeutic efficacy without negatively affecting the patient’s quality of life. Although PTT is emerging as a promising therapeutic approach due to its localized and noninvasive nature, the lack of tumor-targeting properties of photoabsorbers leads to their sporadic distribution, resulting in undesired side effects. By contrast, we demonstrated GNDs@gelatin as a TME-sensitive theranostic nanoplatform that self-assembles *in situ* to exhibit excellent PTT efficacy in both NIR I and II regions with no incidence of metastasis under moderate photothermal temperatures using lower laser power density. Of great interest and importance to these studies, it implicates the predicted metastasis could be modulated by judicious tuning of the physical parameters in PTT. These data suggest a new paradigm of “moderate is better” in the application of nanoparticle-based PTT for maximizing its therapeutic benefits and translational potential.

## Supporting Information Available

Additional experimental details, including the MCH to MPA ratio-dependent morphologies and absorption spectral evolution of GNDs@gelatin; effect of gelatinase activity on cellular uptake of GNDs@gelatin; dot plot of significant gene sets selected by multiple enrichment analysis; Venn diagram of DEGs for making comparison among cell proliferation and cell migration pathway, and tables of differential expression gene sets (PDF)

## Abbreviations

NIR-I/-II: near-infrared I/II
PTT: photothermal therapy
GNDs: gold nanodandelions
PA: photoacoustic
MMP: matrix metalloproteinase
DEGs: differentially expressed genes
MRI: magnetic resonance imaging
CT: computed tomography
PET: positron emission tomography
SPECT: single-photon emission computed tomography
AuNPs: gold nanoparticles
TME: tumor microenvironment
MCH: 6-mercaptohexan-1-ol
MPA: 3-sulfanylpropanoic acid
US: ultrasound
ROI: region of interest
SNR: signal-to-noise ratio
fwhm: full width at half-maximum
ANSI: American National Standards Institute
TEM: transmission electron microscopy
SAM: self-assembled monolayer
FIRDI: Food Industry Research and Development Institute
PI: propidium iodide
CW: continuous wavelength
LSPR: localized surface plasmon resonance
MPE: maximum permissible exposure
BPs: biological processes
MFs: molecule functions
RT-qPCR: quantitative reverse transcription PCR
EMT: epithelial-mesenchymal transition
MDR: multidrug resistance

## References

[ref1] Wong X. Y.; Sena-Torralba A.; Álvarez-Diduk R.; Muthoosamy K.; Merkoçi A. Nanomaterials for nanotheranostics: Tuning their properties according to disease needs. ACS Nano 2020, 14, 2585–627. 10.1021/acsnano.9b08133. 32031781

[ref2] Sivasubramanian M.; Chuang Y. C.; Chen N. T.; Lo L. W. Seeing better and going deeper in cancer nanotheranostics. Int. J. Mol. Sci. 2019, 20, 3490 10.3390/ijms20143490. 31315232 PMC6678689

[ref3] Yoon H. Y.; Jeon S.; You D. G.; Park J. H.; Kwon I. C.; Koo H.; et al. Inorganic nanoparticles for image-guided therapy. Bioconjug Chem. 2017, 28, 124–34. 10.1021/acs.bioconjchem.6b00512. 27788580

[ref4] Moore C.; Jokerst J. V. Strategies for image-guided therapy, surgery, and drug delivery using photoacoustic imaging. Theranostics 2019, 9, 1550–71. 10.7150/thno.32362. 31037123 PMC6485201

[ref5] Attia A. B. E.; Balasundaram G.; Moothanchery M.; Dinish U. S.; Bi R.; Ntziachristos V.; et al. A review of clinical photoacoustic imaging: Current and future trends. Photoacoustics 2019, 16, 100144 10.1016/j.pacs.2019.100144. 31871888 PMC6911900

[ref6] Xu C.; Wang Y.; Wang E.; Yan N.; Sheng S.; Chen J.; et al. Effective eradication of tumors by enhancing photoacoustic-imaging-guided combined photothermal therapy and ultrasonic therapy. Adv. Funct. Mater. 2021, 31, 2009314 10.1002/adfm.202009314.

[ref7] Liu Y.; Bhattarai P.; Dai Z.; Chen X. Photothermal therapy and photoacoustic imaging via nanotheranostics in fighting cancer. Chem. Soc. Rev. 2019, 48, 2053–108. 10.1039/C8CS00618K. 30259015 PMC6437026

[ref8] Cai K.; Zhang W.; Foda M. F.; Li X.; Zhang J.; Zhong Y.; et al. Miniature hollow gold nanorods with enhanced effect for in vivo photoacoustic imaging in the NIR-II window. Small 2020, 16, 2002748 10.1002/smll.202002748. 32780938

[ref9] Lindley S. A.; Zhang J. Z. Bumpy hollow gold nanospheres for theranostic applications: effect of surface morphology on photothermal conversion efficiency. ACS Appl. Nano Mater. 2 2019, 2, 1072–81. 10.1021/acsanm.8b02331.

[ref10] Park J. H.; Dumani D. S.; Arsiwala A.; Emelianov S.; Kane R. S. Tunable aggregation of gold-silica janus nanoparticles to enable contrast-enhanced multiwavelength photoacoustic imaging in vivo. Nanoscale 2018, 10, 15365–70. 10.1039/C8NR03973A. 30083665

[ref11] Zhou J.; Jiang Y.; Hou S.; Upputuri P. K.; Wu D.; Li J.; et al. Compact plasmonic blackbody for cancer theranosis in the near-infrared II window. ACS Nano 2018, 12, 2643–51. 10.1021/acsnano.7b08725. 29438610

[ref12] Xu Y.; Wang X.; Cheng L.; Liu Z.; Zhang Q. High-yield synthesis of gold bipyramids for in vivo CT imaging and photothermal cancer therapy with enhanced thermal stability. Chem. Eng. J. 2019, 378, 122025 10.1016/j.cej.2019.122025.

[ref13] Zhang J.; Ning L.; Zeng Z.; Pu K. Development of second near-infrared photoacoustic imaging agents. Trends Chem. 2021, 3, 305–17. 10.1016/j.trechm.2021.01.002.

[ref14] Cheng P.; Pu K. Activatable phototheranostic materials for imaging-guided cancer therapy. ACS Appl. Nano Mater. 2020, 12, 5286–99. 10.1021/acsami.9b15064. 31730329

[ref15] Cheng X.; Sun R.; Yin L.; Chai Z.; Shi H.; Gao M. Light-triggered assembly of gold nanoparticles for photothermal therapy and photoacoustic imaging of tumors in vivo. Adv. Mater. 2017, 29, 1604894 10.1002/adma.201604894. 27921316

[ref16] Li S.; Lui K. H.; Tsoi T. H.; Lo W. S.; Li X.; Hu X.; et al. pH-responsive targeted gold nanoparticles for in vivo photoacoustic imaging of tumor microenvironments. Nanoscale Adv. 2019, 1, 554–64. 10.1039/C8NA00190A. 36132235 PMC9473232

[ref17] Liu Y.; Yang Z.; Huang X.; Yu G.; Wang S.; Zhou Z.; et al. Glutathione-responsive self-assembled magnetic gold nanowreath for enhanced tumor imaging and imaging-guided photothermal therapy. ACS Nano 2018, 12, 8129–37. 10.1021/acsnano.8b02980. 30001110

[ref18] Zhang Y.; Chang J.; Huang F.; Yang L.; Ren C.; Ma L.; et al. Acid-triggered in situ aggregation of gold nanoparticles for multimodal tumor imaging and photothermal therapy. ACS Biomater Sci. Eng. 2019, 5, 1589–601. 10.1021/acsbiomaterials.8b01623. 33405632

[ref19] Sun M.; Liu F.; Zhu Y.; Wang W.; Hu J.; Liu J.; et al. Salt-induced aggregation of gold nanoparticles for photoacoustic imaging and photothermal therapy of cancer. Nanoscale 2016, 8, 4452–7. 10.1039/C6NR00056H. 26847879

[ref20] Nam J.; La W. G.; Hwang S.; Ha Y. S.; Park N.; Won N.; et al. pH-responsive assembly of gold nanoparticles and “spatiotemporally concerted” drug release for synergistic cancer therapy. ACS Nano 2013, 7, 3388–402. 10.1021/nn400223a. 23530622

[ref21] Isaacson K. J.; Martin Jensen M.; Subrahmanyam N. B.; Ghandehari H. Matrix-metalloproteinases as targets for controlled delivery in cancer: An analysis of upregulation and expression. J. Controlled Release 2017, 259, 62–75. 10.1016/j.jconrel.2017.01.034. PMC5537048 28153760

[ref22] Sawaya R. E.; Yamamoto M.; Gokaslan Z. L.; Wang S. W.; Mohanam S.; Fuller G. N.; et al. Expression and localization of 72 kDa type IV collagenase (MMP-2) in human malignant gliomas in vivo. Clin Exp Metastasis. 1996, 14, 35–42. 10.1007/BF00157684. 8521615

[ref23] Chuang Y. C.; Hsia Y.; Chu C. H.; Lin L. J.; Sivasubramaniana M.; Lo L. W. Precision control of the large-scale green synthesis of biodegradable gold nanodandelions as potential radiotheranostics. Biomater Sci. 2019, 7, 4720 10.1039/C9BM00897G. 31495835

[ref24] Wang Y.; Jhang D. F.; Tsai C. H.; Chiang N. J.; Tsao C. H.; Chuang C. C.; et al. In vivo assessment of hypoxia levels in pancreatic tumors using a dual-modality ultrasound/photoacoustic imaging system. Micromachines 2021, 12, 668 10.3390/mi12060668. 34200388 PMC8229757

[ref25] Leng H.; Wang Y.; Jhang D. F.; Chu T. S.; Tsao C. H.; Tsai C. H.; et al. Characterization of a fiber bundle-based real-time ultrasound/photoacoustic imaging system and its in vivo functional imaging applications. Micromachines 2019, 10, 820 10.3390/mi10120820. 31783545 PMC6953120

[ref26] Cai K.; Zhang W.; Zhang J.; Li H.; Han H.; Zhai T. Design of gold hollow nanorods with controllable aspect ratio for multimodal imaging and combined chemo-photothermal therapy in the second near-Infrared window. ACS Appl. Mater. Interfaces 2018, 10, 36703–10. 10.1021/acsami.8b12758. 30284807

[ref27] Zheng W.; He L. Particle stability in polymer-assisted reverse colorimetric DNA assays. Anal. Bioanal. Chem. 2009, 393, 1305–13. 10.1007/s00216-008-2536-4. 19089415

[ref28] Chuang Y. C.; Li J. C.; Chen S. H.; Liu T. Y.; Kuo C. H.; Huang W. T.; et al. An optical biosensing platform for proteinase activity using gold nanoparticles. Biomaterials 2010, 31, 6087–95. 10.1016/j.biomaterials.2010.04.026. 20471084

[ref29] Panikkanvalappil S. R.; Hooshmand N.; El-Sayed M. A. Intracellular assembly of nuclear-targeted gold nanosphere enables selective plasmonic photothermal therapy of cancer by shifting their absorption wavelength toward near-infrared region. Bioconjug Chem. 2017, 28, 2452–60. 10.1021/acs.bioconjchem.7b00427. 28837765

[ref30] Mei Z.; Gao D.; Hu D.; Zhou H.; Ma T.; Huang L.; et al. Activatable NIR-II photoacoustic imaging and photochemical synergistic therapy of MRSA infections using miniature Au/Ag nanorods. Biomaterials 2020, 251, 120092 10.1016/j.biomaterials.2020.120092. 32388165

[ref31] Raudvere U.; Kolberg L.; Kuzmin I.; Arak T.; Adler P.; Peterson H.; et al. g:Profiler: a web server for functional enrichment analysis and conversions of gene lists (2019 update). Nucleic Acids Res. 2019, 47, W191–W198. 10.1093/nar/gkz369. 31066453 PMC6602461

[ref32] Chuang Y. C.; Chen Y. P.; Wu H. M.; Hsu J. S.; Lo L. W.; Wang Y. M. Matrix metalloproteinase-directed precise targeting and smart drug delivery of biodegradable gold nanodandelions as CT imaging guided anticancer therapy. J. Drug Deliv Sci. Technol. 2022, 74, 103563 10.1016/j.jddst.2022.103563.

[ref33] Schulz R. Intracellular targets of matrix metalloproteinase-2 in cardiac disease: rationale and therapeutic approaches. Annu. Rev. Pharmacol. Toxicol 2007, 47, 211–42. 10.1146/annurev.pharmtox.47.120505.105230. 17129183

[ref34] Wild-Bode C.; Weller M.; Wick W. Molecular determinants of glioma cell migration and invasion. J. Neurosurg. 2001, 94, 978–84. 10.3171/jns.2001.94.6.0978. 11409528

[ref35] Yang K.; Liu Y.; Wang Y.; Ren Q.; Guo H.; Matson J. B.; et al. Enzyme-induced in vivo assembly of gold nanoparticles for imaging-guided synergistic chemo-photothermal therapy of tumor. Biomaterials 2019, 223, 119460 10.1016/j.biomaterials.2019.119460. 31513993 PMC6764864

[ref36] Chen H.; Shao L.; Ming T.; Sun Z.; Zhao C.; Yang B.; et al. Understanding the photothermal conversion efficiency of gold nanocrystals. Small 2010, 6, 2272–80. 10.1002/smll.201001109. 20827680

[ref37] Wang X.; Zhu M.; Sun Y.; Fu W.; Gu Q.; Zhang C.; Zhang Y.; Dai Y.; Sun Y. A new insight of the photothermal effect on the highly efficient visible-light-driven photocatalytic performance of novel-designed TiO_2_ rambutan-like microspheres. Part. Part. Syst. Charact. 2016, 33, 140–149. 10.1002/ppsc.201500139.

[ref38] Huang C. P.; Lin J.; Li W.; Rong P.; Wang Z.; Wang S.; et al. Biodegradable gold nanovesicles with an ultrastrong plasmonic coupling effect for photoacoustic imaging and photothermal therapy. Angew. Chem., Int. Ed. Engl. 2013, 52, 13958–64. 10.1002/anie.201308986. 24318645 PMC4058316

[ref39] Hessel M.; Pattani V. P.; Rasch M.; Panthani M. G.; Koo B.; Tunnell J. W.; et al. Copper selenide nanocrystals for photothermal therapy. Nano Lett. 2011, 11, 2560–6. 10.1021/nl201400z. 21553924 PMC3111000

[ref40] Bear A. S.; Kennedy L. C.; Young J. K.; Perna S. K.; Almeida J. P. M.; Lin A. Y.; et al. Elimination of metastatic melanoma using gold nanoshell-enabled photothermal therapy and adoptive T cell transfer. PloS One 2013, 8, e69073 10.1371/journal.pone.0069073. 23935927 PMC3720863

[ref41] Paholak H. J.; Stevers N. O.; Chen H.; Burnett J. P.; He M.; Korkaya H.; et al. Elimination of epithelial-like and mesenchymal-like breast cancer stem cells to inhibit metastasis following nanoparticle-mediated photothermal therapy. Biomaterials 2016, 104, 145–57. 10.1016/j.biomaterials.2016.06.045. 27450902 PMC5680543

[ref42] Zhang Y.; Zhan X.; Xiong J.; Peng S.; Huang W.; Joshi R.; et al. Temperature-dependent cell death patterns induced by functionalized gold nanoparticle photothermal therapy in melanoma cells. Sci. Rep. 2018, 8, 8720 10.1038/s41598-018-26978-1. 29880902 PMC5992202

[ref43] Chen W.; Wang X.; Zhao B.; Zhang R.; Xie Z.; He Y.; et al. CuS-MnS2 nano-flowers for magnetic resonance imaging guided photothermal/photodynamic therapy of ovarian cancer through necroptosis. Nanoscale. 2019, 11, 12983–9. 10.1039/C9NR03114F. 31264665

[ref44] Zhao H.; Chen H.; Guo Z.; Zhang W.; Yu H.; Zhuang Z.; et al. In situ photothermal activation of necroptosis potentiates black phosphorusmediated cancer photo-immunotherapy. Chem. Eng. J. 2020, 394, 124314 10.1016/j.cej.2020.124314.

[ref45] Gao G.; Sun X.; Liang G. Nanoagent-promoted mild-temperature photothermal therapy for cancer treatment. Adv. Funct. Mater. 2021, 31, 2100738 10.1002/adfm.202100738.

