# Supplementary material for: Photothermal Temperature-Modulated Cancer Metastasis Harnessed Using Proteinase-Triggered Assembly of Near-Infrared II Photoacoustic/Photothermal Nanotheranostics
Source: ACS Appl Mater Interfaces. 2024 Jul 24;16(31):40611–27. doi: 10.1021/acsami.4c07173 (PMC11310908; doi:10.1021/acsami.4c07173)
Supplement: Supplementary file 1 — am4c07173_si_001.pdf [file am4c07173_si_001.pdf]

Supporting Information

Photothermal Temperature-Modulated Cancer Metastasis Harnessed Using Proteinase-Triggered Assembly of Near-Infrared II Photoacoustic/Photothermal Nanotheranostics

Yao-Chen Chuang [a, b], Yu Hsia [a, c], Chia-Hui Chu [a], Maharajan Sivasubramanian [a], Fang-Chi Hsu [d], Hsin-Lun Lee [b, e], Jeng Fong Chiou [b, e], Hui-Ju Ch'ang [f], Lun-De Liao [a, *], and Leu-Wei Lo [a, *]

[a.] Institute of Biomedical Engineering and Nanomedicine, National Health Research Institutes, Zhunan, Miaoli 35053, Taiwan

[b.] Department of Radiation Oncology, Taipei Medical University Hospital, Taipei 110301, Taiwan

[c.] Institute of Biotechnology, National Tsing Hua University, Hsinchu 30013, Taiwan

[d.] The Ph.D. Program for Translational Medicine, College of Medical Science and Technology, Taipei Medical University and Academia Sinica, Taipei 110301, Taiwan

[e.] Department of Radiology, School of Medicine, College of Medicine, Taipei Medical University, Taipei 110301, Taiwan

[f.] National Institute of Cancer Research, National Health Research Institutes, Zhunan, Miaoli 35053, Taiwan

* Author for correspondence:

Leu-Wei Lo, Ph.D.

Institute of Biomedical Engineering and Nanomedicine, National Health Research Institutes, Zhunan, Miaoli 35053, Taiwan.

Tel: 886-37-206166 # 37115; Fax: 886-37-586440

E-mail: lwlo@nhri.edu.tw

Lun-De Liao, Ph.D.

Institute of Biomedical Engineering and Nanomedicine, National Health Research Institutes, Zhunan, Miaoli 35053, Taiwan.

Tel: 886-37-206166 # 37125; Fax: 886-37-586440

E-mail: ldliao@nhri.edu.tw

Author Contributions: Leu-Wei Lo conceptualized and supervised the research; Lun-De Liao designed the photoacoustic experiments. Yao-Chen Chuang, Yu Hsia and Chia-Hui Chu performed both in vitro and in vivo photothermal and photoacoustic experiments and analyzed the data. Yao-Chen Chuang, Maharajan Sivasubramanian, Fang-Chi Hsu, Hsin-Lun Lee, Jeng Fong Chiou, Hui-Ju Ch'ang, and Leu-Wei Lo wrote the paper. All the authors contributed to the article and approved the submitted version.

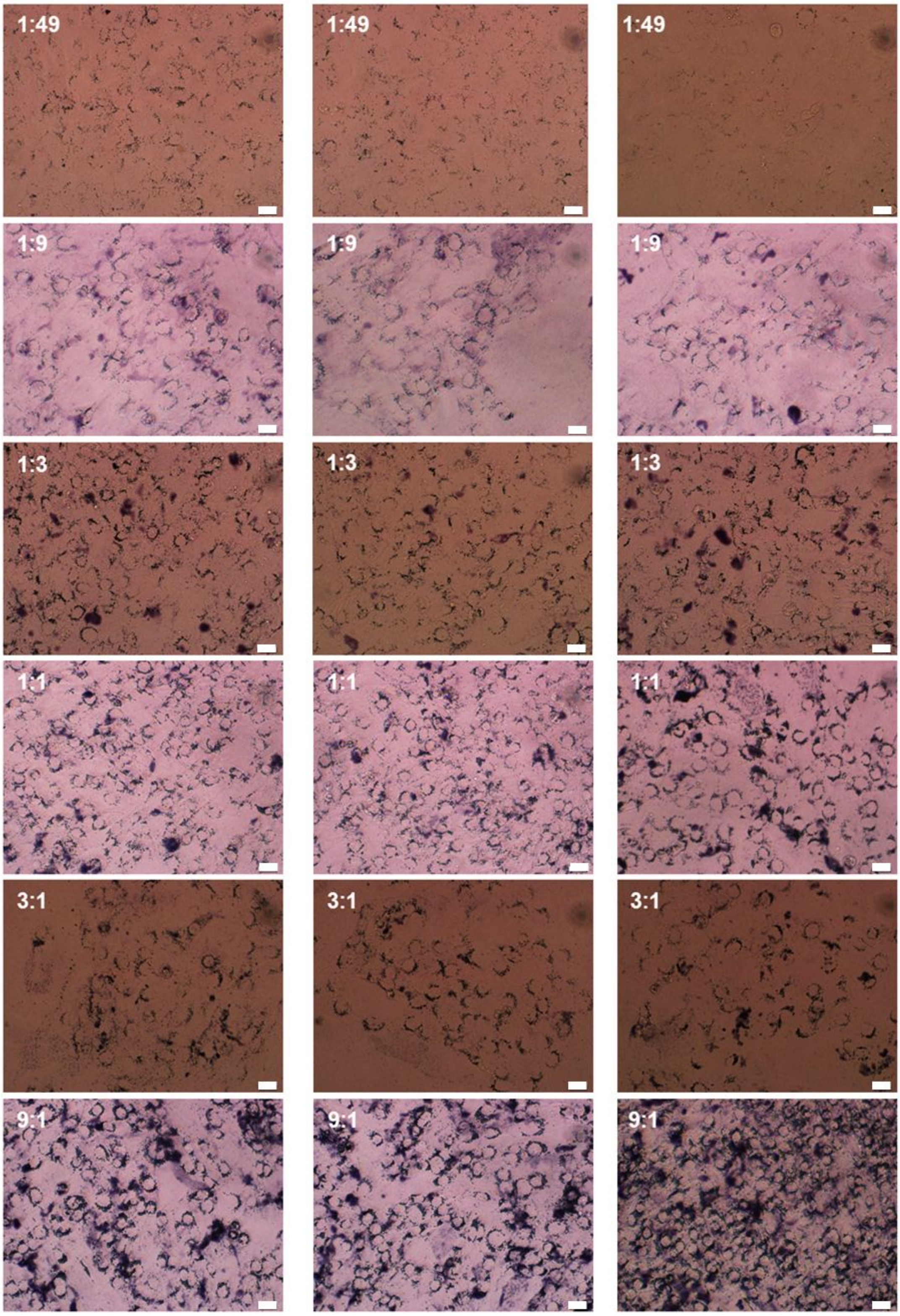

Figure S1. The MCH to MPA ratio-dependent morphologies and absorption spectral evolution of GNDs@gelatin $_{X:Y}$. The optical microscopy images delineate

the uptake efficiencies of GNDs@gelatin $_{X:Y}$ by C6 cells at different MCH:MPA ratios. All scale bars are 20 μm.

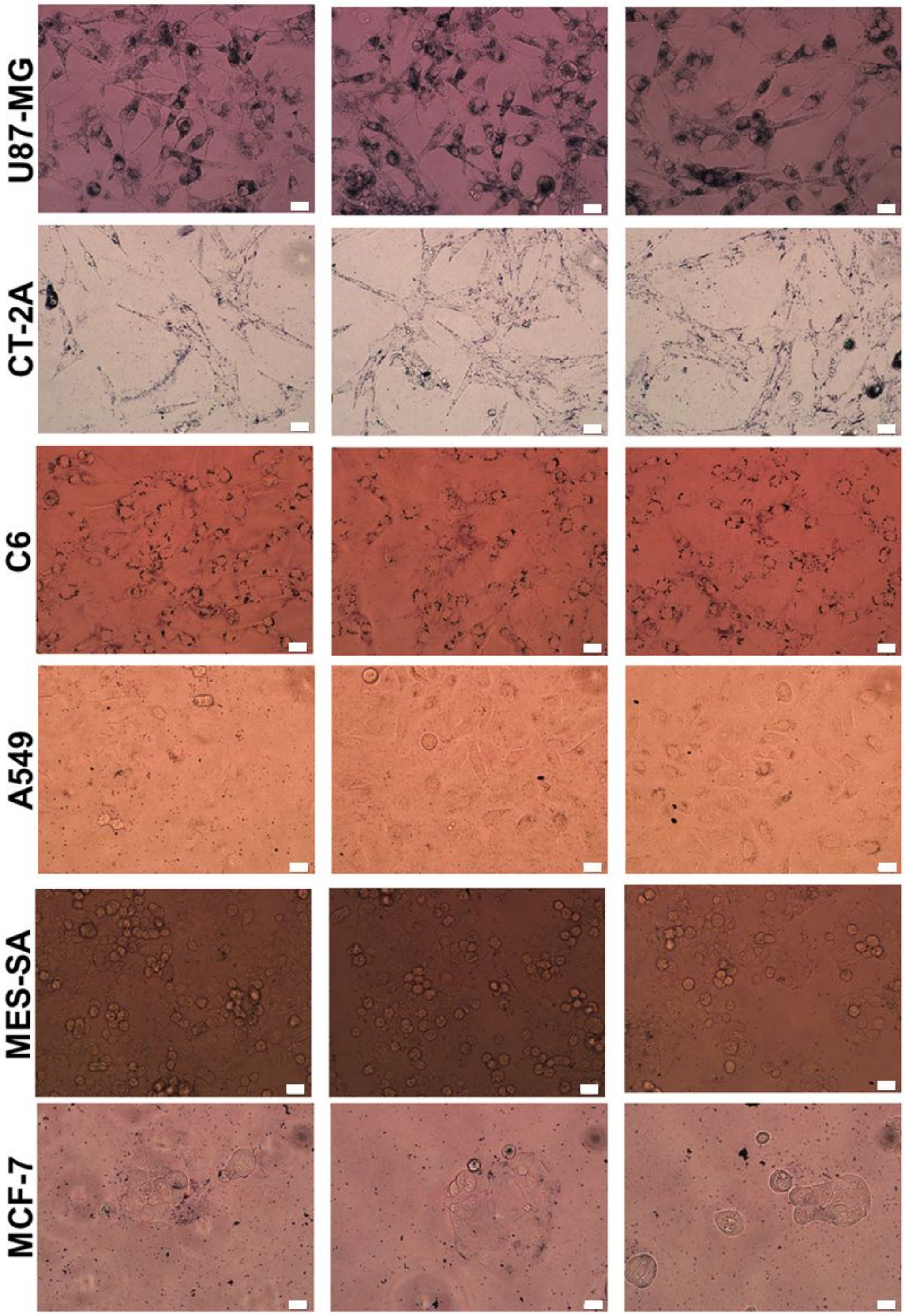

Figure S2. Effects of gelatinase activity on cellular uptake of GNDs@gelatin.

Differential optical microscopy images of U87-MG; CT-2A; C6; A549; MES-SA and MCF-7 cells treated with GNDs@gelatin for 24 h. All scale bars are 20 μm.

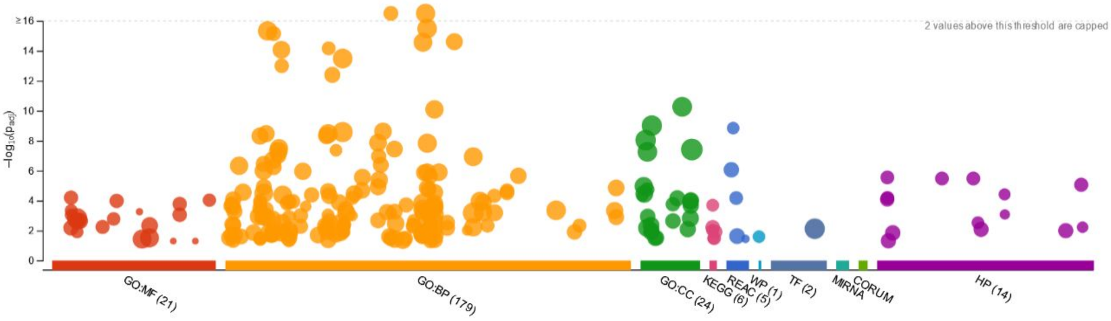

Figure S3. The dot plot of significant gene sets selected by multiple enrichment analysis.

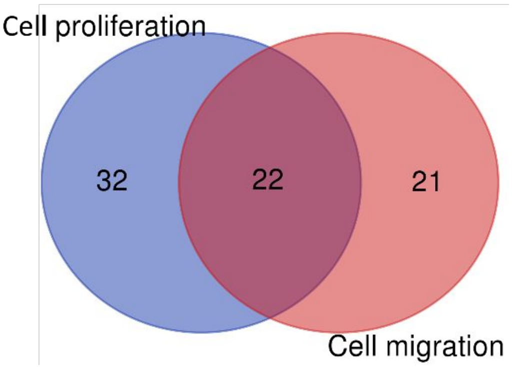

Figure S4. A Venn diagram of DEGs for making comparison among Cell proliferation and cell migration pathway.

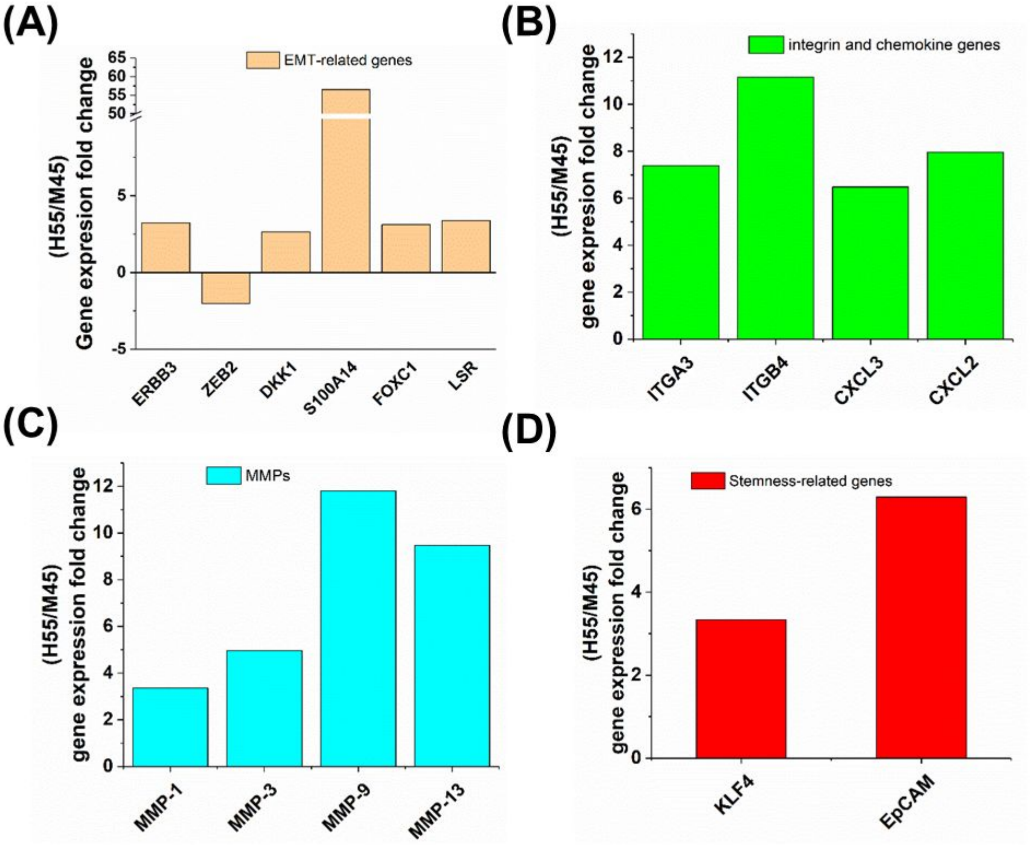

Figure S5. Quantitative microarray analysis of (A) EMT- related genes, (B) integrin and chemokine gene, (C) MMPs and (D) stemness- related genes expression fold change between high temperature H55 and mild temperature M45 groups two days after treatment.

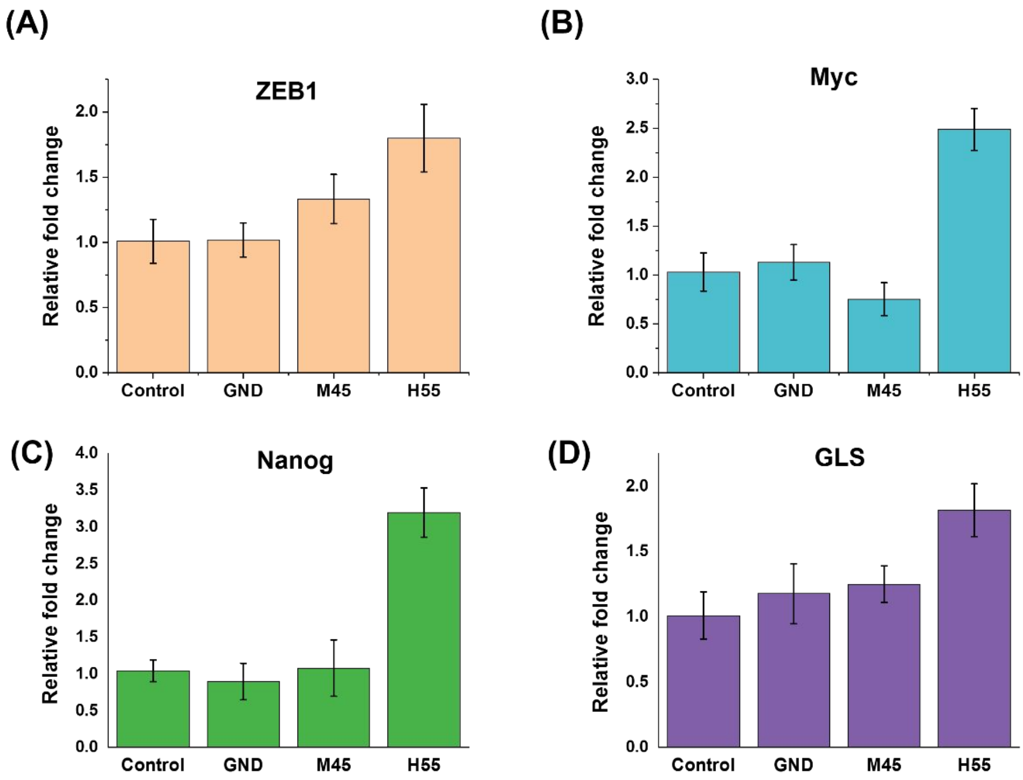

Figure S6. RT-qPCR analysis of (A) EMT- related gene, and (B-D) stemness- related genes expression fold change between high temperature H55 and mild temperature M45 groups 24 h after GND@gelatin PTT treatment.

Table S1. The table of differential expression gene sets

Cluster	adj.Pval	nGenes	Pathways
**A**	8.66E-17	41	Response to pheromone
	8.66E-17	48	Skin development
	1.29E-14	32	Keratinocyte differentiation
	1.68E-14	22	Keratinization
	2.28E-13	45	Epidermis development
	9.85E-13	36	Epidermal cell differentiation
	2.61E-05	50	Epithelial cell differentiation
	0.00031857	17	Membrane lipid biosynthetic process
	0.00116633	20	Membrane lipid metabolic process

	0.00116633	17	Sphingolipid metabolic process
	0.00116633	70	Epithelium development
	0.00129837	15	Molting cycle
	0.00129837	15	Hair cycle
**B**	5.54E-32	35	Skin development
	8.29E-29	21	Keratinization
	3.12E-28	26	Keratinocyte differentiation
	5.07E-27	32	Epidermis development
	1.40E-26	28	Epidermal cell differentiation
	1.42E-19	34	Epithelial cell differentiation
	1.48E-17	41	Epithelium development
	5.33E-14	46	Tissue development
	1.55E-10	8	Establishment of skin barrier
	5.51E-10	9	Intermediate filament-based process
	7.15E-10	8	Regulation of water loss via skin
	3.77E-09	7	Intermediate filament organization
	4.25E-09	9	Multicellular organismal water homeostasis
	1.30E-08	9	Water homeostasis
**C**	2.66E-07	92	Immune system process
	5.79E-07	117	Response to stress
	5.79E-07	66	Immune response
	6.80E-07	64	Defense response
	3.19E-05	36	Inflammatory response
	4.06E-05	36	Innate immune response
	9.26E-05	43	Cell activation
	0.00010637	43	Defense response to other organism
	0.00011815	86	Response to external stimulus
	0.00011815	54	Response to biotic stimulus
	0.00011815	53	Response to other organism
	0.00012018	56	Biological process involved in interspecies interaction between organisms
	0.00016537	7	DNA replication initiation
	0.00038631	5	Response to macrophage colony-stimulating factor
	0.00038631	5	Cellular response to macrophage colony-stimulating factor stimulus
**D**	1.21E-06	152	Response to organic substance

	1.28E-06	159	Response to stress
	3.41E-06	9	Antigen processing and presentation of peptide or polysaccharide antigen via MHC class II
	8.96E-06	59	Carbohydrate derivative metabolic process
	8.96E-06	87	Small molecule metabolic process
	8.96E-06	135	Cellular response to chemical stimulus
	3.41E-05	121	Response to external stimulus
	4.80E-05	31	Wound healing
	5.08E-05	7	Antigen processing and presentation of peptide antigen via MHC class II
	5.75E-05	37	Response to wounding
	5.75E-05	112	Immune system process
	5.75E-05	42	Muscle structure development
	6.94E-05	39	Carbohydrate derivative biosynthetic process
	7.13E-05	54	Response to cytokine
	7.13E-05	109	Cellular response to organic substance

Table S2. The table of significant pathways.

term_name	term_id	Adj_p_Value	term_size	query_siaze	intersection_size
immune system process	GO:0002376	7.53E-07	2794	279	64
cell population proliferation	GO:0008283	3.03E-07	2081	279	54
movement of cell or subcellular component	GO:0006928	1.54E-06	2112	279	53
cell adhesion	GO:0007155	5.04E-09	1411	279	46
biological adhesion	GO:0022610	6.75E-09	1423	279	46
cell motility	GO:0048870	1.79E-06	1684	279	46
regulation of cell population proliferation	GO:0042127	5.91E-06	1749	279	46
cell migration	GO:0016477	1.56E-06	1498	279	43
regulation of immune system process	GO:0002682	3.73E-05	1483	279	40
response to cytokine	GO:0034097	4.55E-05	1140	279	34
regulation of cell motility	GO:2000145	1.85E-05	989	279	32
cell-cell adhesion	GO:0098609	2.88E-06	864	279	31
cellular response to cytokine stimulus	GO:0071345	0.00016091	1029	279	31
regulation of cell migration	GO:0030334	5.98E-05	930	279	30
positive regulation of cell population proliferation	GO:0008284	0.002111826	1040	279	29
inflammatory response	GO:0006954	1.55E-05	723	279	27
regulation of cell adhesion	GO:0030155	2.98E-05	746	279	27
response to wounding	GO:0009611	1.27E-07	537	279	26

wound healing	GO:0042060	1.33E-07	379	279	22
epithelial cell proliferation	GO:0050673	2.21E-05	455	279	21
positive regulation of cell motility	GO:2000147	0.001520895	586	279	21
cytokine-mediated signaling pathway	GO:0019221	4.94E-05	433	279	20
epithelium migration	GO:0090132	2.60E-05	297	279	17
tissue migration	GO:0090130	3.49E-05	303	279	17
epithelial cell migration	GO:0010631	0.000141608	294	279	16
protein binding	GO:0005515	0.000666713	10433	284	156
transition metal ion binding	GO:0046914	0.005972919	1083	284	29
lipid binding	GO:0008289	0.002908902	822	284	25
calcium ion binding	GO:0005509	0.00306962	719	284	23
endopeptidase inhibitor activity	GO:0004866	7.64E-05	217	284	14
peptidase inhibitor activity	GO:0030414	0.000126	226	284	14
endopeptidase regulator activity	GO:0061135	0.000201	235	284	14
peptidase regulator activity	GO:0061134	0.001047	270	284	14
iron ion binding	GO:0005506	0.002164	211	284	12
immune receptor activity	GO:0140375	0.000109669	130	284	11
cytokine binding	GO:0019955	0.001932	141	284	10
cytokine receptor activity	GO:0004896	0.000531	95	284	9
cysteine-type endopeptidase inhibitor activity	GO:0004869	0.001039	77	284	8
interleukin-20 binding	GO:0042015	0.000601	3	284	3
Cytokine-cytokine receptor interaction	KEGG:04060	0.007639	285	146	15

Proteoglycans in cancer	KEGG:05205	0.014482	204	146	12
Viral protein interaction with cytokine and cytokine receptor	KEGG:04061	0.000242	91	146	10
Estrogen signaling pathway	KEGG:04915	0.037613	134	146	9
PPAR signaling pathway	KEGG:03320	0.010803	89	146	8
VEGF signaling pathway	KEGG:04370	0.03699	58	146	6
African trypanosomiasis	KEGG:05143	0.032724	37	146	5

Notes and references

1. Wang Y, Jhang DF, Tsai CH, Chiang NJ, Tsao CH, Chuang CC, Chen LT, Chang WSW, Liao LD. *In vivo* assessment of hypoxia levels in pancreatic tumors using a dual-modality ultrasound/photoacoustic imaging system. *Micromachines* 2021, **12**:668.

2. Leng H, Wang Y, Jhang DF, Chu TS, Tsao CH, Tsai CH, Giamundo S, Chen YY, Liao KW, Chuang CC, Ger TR, Chen LT, Liao LD. Characterization of a fiber bundle-based real-time ultrasound/photoacoustic imaging system and its in vivo functional imaging applications. *Micromachines* 2019, **10**:820.

3. Cai K, Zhang W, Zhang J, Li H, Han H, Zhai T. Design of gold hollow nanorods with controllable aspect ratio for multimodal imaging and combined chemo-photothermal therapy in the second near-Infrared window. *ACS Appl. Mater. Interfaces* 2018, **10**:36703-10.
